# A Nitric Oxide Regulated Small RNA Controls Expression of Genes Involved in Redox Homeostasis in *Bacillus subtilis*

**DOI:** 10.1371/journal.pgen.1004957

**Published:** 2015-02-02

**Authors:** Sylvain Durand, Frédérique Braun, Efthimia Lioliou, Cédric Romilly, Anne-Catherine Helfer, Laurianne Kuhn, Noé Quittot, Pierre Nicolas, Pascale Romby, Ciarán Condon

**Affiliations:** 1 CNRS FRE 3630 (affiliated with Univ. Paris Diderot, Sorbonne Paris Cité), Institut de Biologie Physico-Chimique, Paris, France; 2 Architecture et Réactivité de l’ARN, Université de Strasbourg, CNRS, IBMC, Strasbourg, France; 3 Plateforme Protéomique Esplanade, IBMC, Strasbourg, France; 4 Mathématique Informatique et Génome, INRA UR1077, Jouy en Josas, France; The University of Texas Health Science Center at Houston, UNITED STATES

## Abstract

RsaE is the only known *trans*-acting small regulatory RNA (sRNA) besides the ubiquitous 6S RNA that is conserved between the human pathogen *Staphylococcus aureus* and the soil-dwelling Firmicute *Bacillus subtilis*. Although a number of RsaE targets are known in *S. aureus*, neither the environmental signals that lead to its expression nor its physiological role are known. Here we show that expression of the *B. subtilis* homolog of RsaE is regulated by the presence of nitric oxide (NO) in the cellular milieu. Control of expression by NO is dependent on the ResDE two-component system in *B. subtilis* and we determined that the same is true in *S. aureus*. Transcriptome and proteome analyses revealed that many genes with functions *r*elated to *o*xidative *s*tress and oxidation-reduction reactions were up-regulated in a *B. subtilis* strain lacking this sRNA. We have thus renamed it RoxS. The prediction of RoxS-dependent mRNA targets also suggested a significant enrichment for mRNAs related to respiration and electron transfer. Among the potential direct mRNA targets, we have validated the *ppnKB* mRNA, encoding an NAD^+^/NADH kinase, both *in vivo* and *in vitro*. RoxS controls both translation initiation and the stability of this transcript, in the latter case *via* two independent pathways implicating RNase Y and RNase III. Furthermore, RNase Y intervenes at an additional level by processing the 5′ end of the RoxS sRNA removing about 20 nucleotides. Processing of RoxS allows it to interact more efficiently with a second target, the *sucCD* mRNA, encoding succinyl-CoA synthase, thus expanding the repertoire of targets recognized by this sRNA.

## Author Summary

Bacteria have evolved various strategies to continually monitor the redox state of the internal and external environments to prevent cell damage and/or to protect them from host defense mechanisms. These signals modify the expression of genes, allowing bacteria to adapt to altered redox environments and to maintain homeostasis. Studies in *Enterobacteriaceae* have shown that sRNAs play central roles in adaptation to oxidative stress. We show here that the conserved sRNA, RoxS is induced by the presence of nitric oxide (NO) in the medium, through the ResDE and SrrAB two-component systems of *Bacillus subtilis* and *Staphylococcus aureus*, respectively. *B. subtilis* RoxS regulates functions related to oxidation-reduction reactions and acts as an antisense RNA to control translation initiation and the degradation of *ppnKB* mRNA, encoding an NAD+/NADH kinase. Interestingly, RNase Y processes the 5′ end of the RoxS sRNA leading to a truncated sRNA that in turn interacts more efficiently with a second target, the *sucCD* mRNA, encoding succinyl-CoA synthase. Taken together this work shows that RoxS is part of a complex regulatory network that allows the cell to sense and respond to redox perturbations, and revealed a novel process that allows an expansion of the repertoire of sRNA targets.

## Introduction

Small regulatory RNAs (sRNA) have been shown to play key roles in the regulation of a wide variety of cellular processes in bacteria, including stress responses, environmental signaling and virulence [1,2]. They generally regulate at the post-transcriptional level by altering mRNA translation or stability. Most sRNAs identified to date base pair with the 5’ untranslated region (5’-UTR) and alter ribosome binding to the mRNA. Changes in translation rates often have indirect consequences for mRNA stability as ribosomes can shield mRNA from attack by ribonucleases. A number of sRNAs have also been shown to directly affect mRNA stability without altering translation initiation rates through interactions with the 5’-UTR, the 3’-UTR or the coding sequence [3,4,5,6].

Although bacterial sRNAs have been studied most extensively in *Escherichia coli* and closely related organisms, the link to virulence has led to the identification and characterization of sRNAs in a wide range of both Gram-negative and Gram-positive bacterial pathogens. The Gram-positive model organism *Bacillus subtilis* trails conspicuously behind in these efforts, where only two *trans*-acting sRNAs, SR1 and FsrA, have been studied in detail [7–11]. The RNA chaperone Hfq has been shown to play a key role in sRNA association with its mRNA target in Proteobacteria. However, its role in Firmicutes seems to be less evident [7,8,12–14], suggesting that alternative RNA chaperones remain to be discovered in these organisms. Furthermore, there are important differences in the mRNA degradation machineries and pathways of these two bacterial clades, most notably the widespread occurrence of a 5’-3’ exoribonuclease activity provided by RNase J in the Firmicutes and the ability of stalled ribosomes to protect long stretches of downstream mRNA from ribonucleolytic attack [15,16]. The RNases involved in the regulation of mRNA stability by sRNAs in the Firmicutes have not been identified in many cases.

RsaE was first discovered in *Staphylococcus aureus* as a member of a family of sRNAs that contain multiple C-rich regions (CRR) that can potentially pair with the G-rich Shine Dalgarno (SD) sequences of ribosome binding sites to inhibit translation [17]. RsaE shows some strain-dependence in its expression patterns [17,18], but in all tested clinical isolates expression of RsaE was maximal during mid-exponential growth and declined in late-exponential/pre-stationary phase [19]. Expression of RsaE in *S. aureus* strain RN6390 was activated by the *agr* quorum sensing system that plays a key role in *S. aureus* virulence [17] and was further shown to be induced by both oxidative stress and high salt conditions [17,18]. Transcriptome and proteome analysis of RsaE deletion strains or overexpressing strains pointed to a role for *S. aureus* RsaE in governing the expression of genes involved in central metabolism, notably folate metabolism and the TCA cycle [17,18].

**Figure 1:**
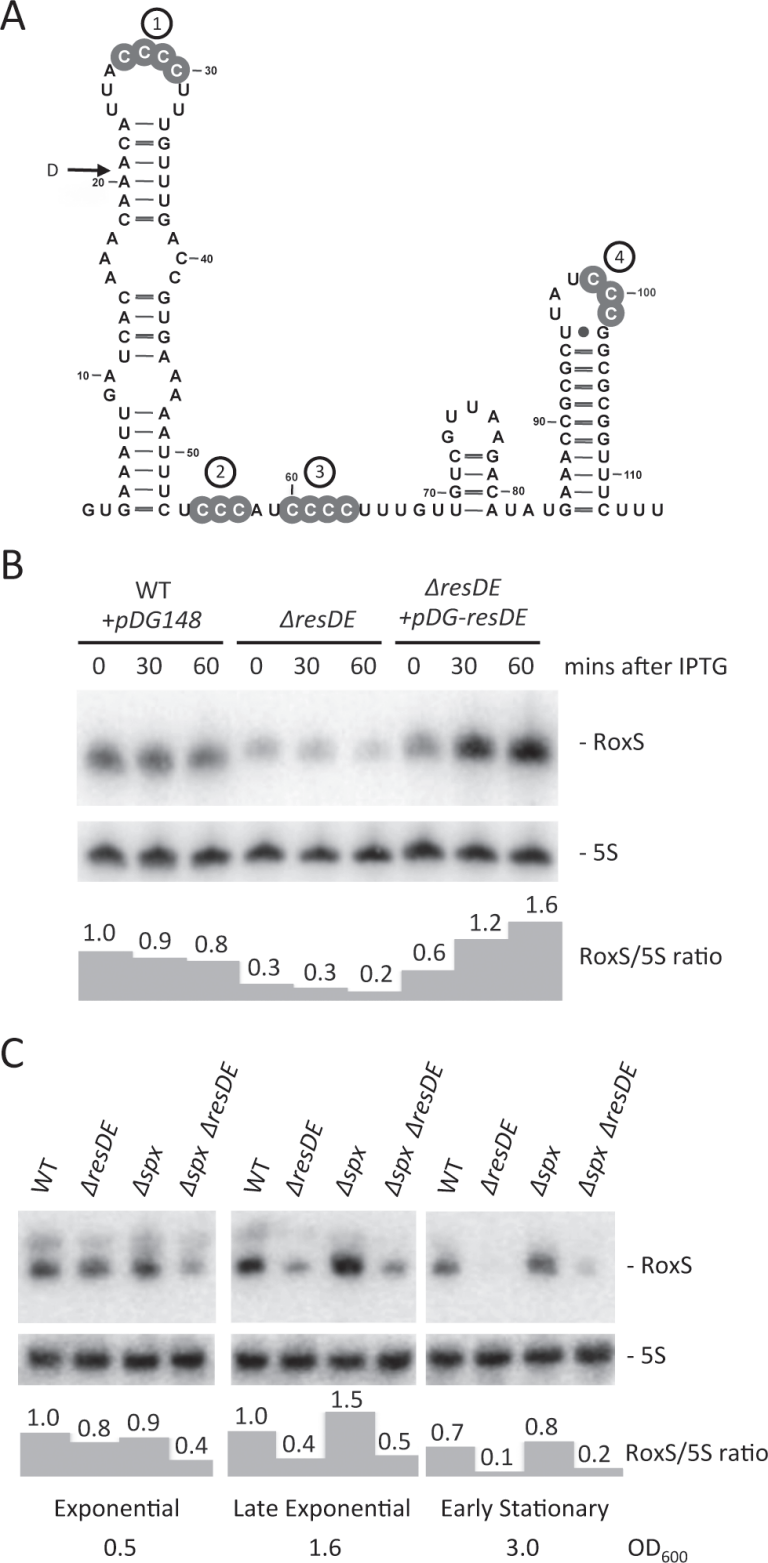
Regulation of RoxS expression by the ResDE two-component system. (A) Consensus secondary structure of RoxS generated with the help of LocaRNA (http://rna.informatik.uni-freiburg.de/LocARNA/). Conserved groups of C-residues are shown in grey circles and are presented as numbered C-rich regions (CRR1-4). The 5’ end of the degradation intermediate (D) observed in the *ΔrnjA* strain is indicated. (B) Northern blot showing RoxS expression in wild-type (WT), a *ΔresDE* mutant and a *ΔresDE* strain complemented with a plasmid expressing *resDE* under IPTG control. Strains used were CCB195 (WT + pDG148), CCB310 (*ΔresDE)* and CCB503 (*ΔresDE* + pDG-resDE). Total RNA was isolated at different times after the addition of 1 mM IPTG to liquid cultures and the blot probed with oligo CC875 (S4 Table). 5S rRNA was probed as a loading control (oligo HP246). RoxS/5S ratios normalized to the WT strain at T0 are presented under the autoradiogram. (C) Northern blot showing RoxS expression in wild-type (WT), CCB310 (*ΔresDE*), CCB628 (*Δspx*) and CCB629 (*ΔresDE Δspx*) mutant strains in cells growing in rich medium in different growth phases. RoxS/5S ratios normalized to the WT strain in exponential phase (OD_600_ = 0.5) are presented under the autoradiogram.

RsaE is highly conserved between *Bacillus* and *Staphylococcus* species at both the primary sequence and predicted secondary structure level [17] (Fig. 1A). The two best-studied representatives of these groups, *B. subtilis* and *S. aureus*, occupy very different ecological niches, the soil and the mammalian skin and respiratory tract, respectively. In these environments, both organisms frequently encounter nitric oxide (NO), a key signaling molecule in both bacteria and eukaryotes (for review, see [20]). Indeed NO, which is toxic at high doses through the production of reactive nitrogen species (RNS), is produced primarily by denitrifying bacteria in the soil and by macrophages in the mammalian host, but some species, notably *Bacilli, Staphylococci* and *Streptomyces*, can also synthesize NO via bacterial NO synthases (bNOS) [21]. NO has been shown in a number of bacteria to provide protection from oxidative stress, provoked either by peroxide [22,23,24] or antibiotics [25,26]. The beneficial effects of NO can also be shared between bacteria and their hosts; NO produced by *B. subtilis* in the intestine of *C. elegans* has been shown to increase the lifespan of the nematode [27]. Despite its importance as both a signaling and potentially stress-inducing molecule, no bacterial sRNA that responds to NO levels has been identified to date. Given that *B. subtilis* is a non-pathogenic organism that occupies a very different niche to *S. aureus*, we were curious as to the physiological role and the targets of this sRNA in *B. subtilis*. We found that expression of RsaE, which we have renamed RoxS in *B. subtilis* for related to oxidative stress, is induced by NO in both *B. subtilis* and *S. aureus*. Despite their similarity in sequence and regulation in the two organisms, the genes affected by deletion of this sRNA are mostly different. Our data illustrate how the functions of a highly conserved sRNA have evolved in distantly related bacteria.

## Results

### RoxS expression is induced by the response regulator ResD in response to increased NO levels in *B. subtilis*

The chromosomal context of the *S. aureus rsaE* and *B. subtilis roxS* genes is very similar and, interestingly, many of the genes have functions related to redox homeostasis or show increased expression under conditions of diamide or peroxide-induced oxidative stress in *B. subtilis* (S1 Fig.) [28]. An alignment of the homologous *roxS/rsaE* genes from several *Bacilli* and *Staphylococci* showed significant sequence conservation in the promoter region (S2 Fig.). An examination of a conserved 8-nucleotide (nt) sequence around position −65 suggested that ResD, the response regulator of the two-component system (TCS) ResDE, that is sensitive to both O_2_ and NO levels [29,30], might recognize this promoter region. Indeed, the sequence upstream of the *roxS* promoter is highly similar to the validated ResD binding site found upstream of the *yclJ* gene [31]. We therefore tested whether the expression of RoxS was altered in a mutant lacking the ResDE TCS. In mid-log phase, a ResDE deletion strain showed a three to four-fold decrease in RoxS expression and this effect was complemented by a plasmid expressing ResDE under IPTG control (Fig. 1B), indicating that ResD is an activator of RoxS transcription. The effect of the ResDE deletion was even stronger as the cells progressed towards stationary phase, confirming its importance as a regulator of RoxS expression (Fig. 1C). In agreement with our data, a recent chromatin immunoprecipitation study has shown a ResD binding at this location of the *B. subtilis* chromosome [32]. In contrast, the thiol specific oxidative stress regulator Spx, also shown to bind in this region [33], had little effect on RoxS expression under the conditions tested.

**Figure 2:**
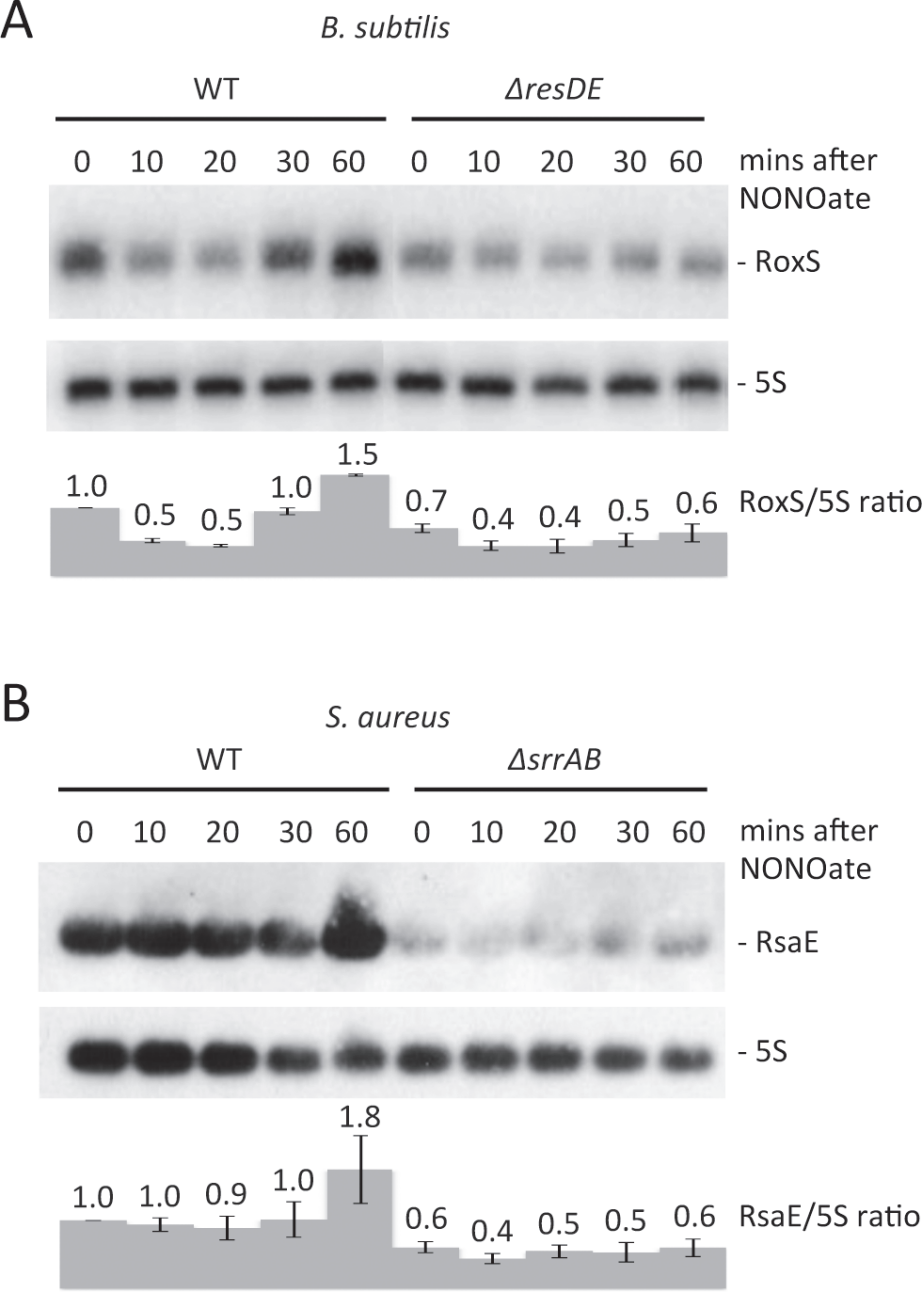
Nitric oxide dependent expression of RoxS in *B. subtilis* and *S. aureus*. Northern blots showing expression of RoxS in (A) wild type (WT) and CCB310 (*ΔresDE*) *B. subtilis* cells and (B) wild type (WT; HG001) and HG001-*ΔsrrAB S. aureus* cells at times after the addition of 100 μM spermine NONOate to cultures growing in rich medium. 5S rRNA was probed as a loading control. RoxS/5S ratios normalized to the WT strain at T0 are presented under each autoradiogram, with standard errors as shown.

The membrane-bound ResE sensor kinase responds to either decreased dissolved O_2_ or increased NO levels [34] by a mechanism that is still not completely understood. It then activates the ResD response regulator through phosphorylation. We tested the effect of NO on RoxS expression by adding spermine NONOate to growing cultures. Spermine NONOate dissolves at neutral pH with a half-life of about 39 mins to produce NO. Expression of RoxS decreased slightly before increasing to a peak 30 mins to 1 h after addition of spermine NONOate (Fig. 2A). Although RoxS expression also decreased slightly upon addition of spermine NONOate to the *ΔresDE* mutant strain, no significant increase was observed after 1 h of incubation. Thus, the NO-dependent induction of RoxS expression depends on the ResDE TCS.

### RsaE expression is also NO and ResDE/SrrAB-dependent in *S. aureus*

Given the strong conservation of the predicted ResD binding site upstream of RsaE in *Staphylococci*, we asked whether expression of RsaE was subjected to a similar regulation in *S. aureus*. The *S. aureus* homolog of the ResDE is called SrrAB and this TCS is also known to respond to low O_2_ levels and NO [30]. The effect of NO on RsaE expression was also tested by adding spermine NONOate under identical conditions to those described for *B. subtilis*. As for *B. subtilis* RoxS, we observed a weak but significant increase in RsaE expression about 1 h after the addition of spermine-NONOate to the growth medium in the wild-type (WT) strain (Fig. 2B). As anticipated, expression of RsaE was significantly lower in steady state conditions in the *srrAB* mutant, suggesting that SrrA is an activator of RsaE transcription. Furthermore, expression of RsaE was no longer induced by the addition of spermine-NONOate in this strain, clearly showing that the induction of RsaE by NO is dependent on the SrrAB TCS. Therefore, the signaling pathway and the expression of *S. aureus* RsaE and *B. subtilis* RoxS have been maintained during evolution.

### *B. subtilis* strains lacking RoxS show increased expression of genes with redox-related functions

**Figure 3:**
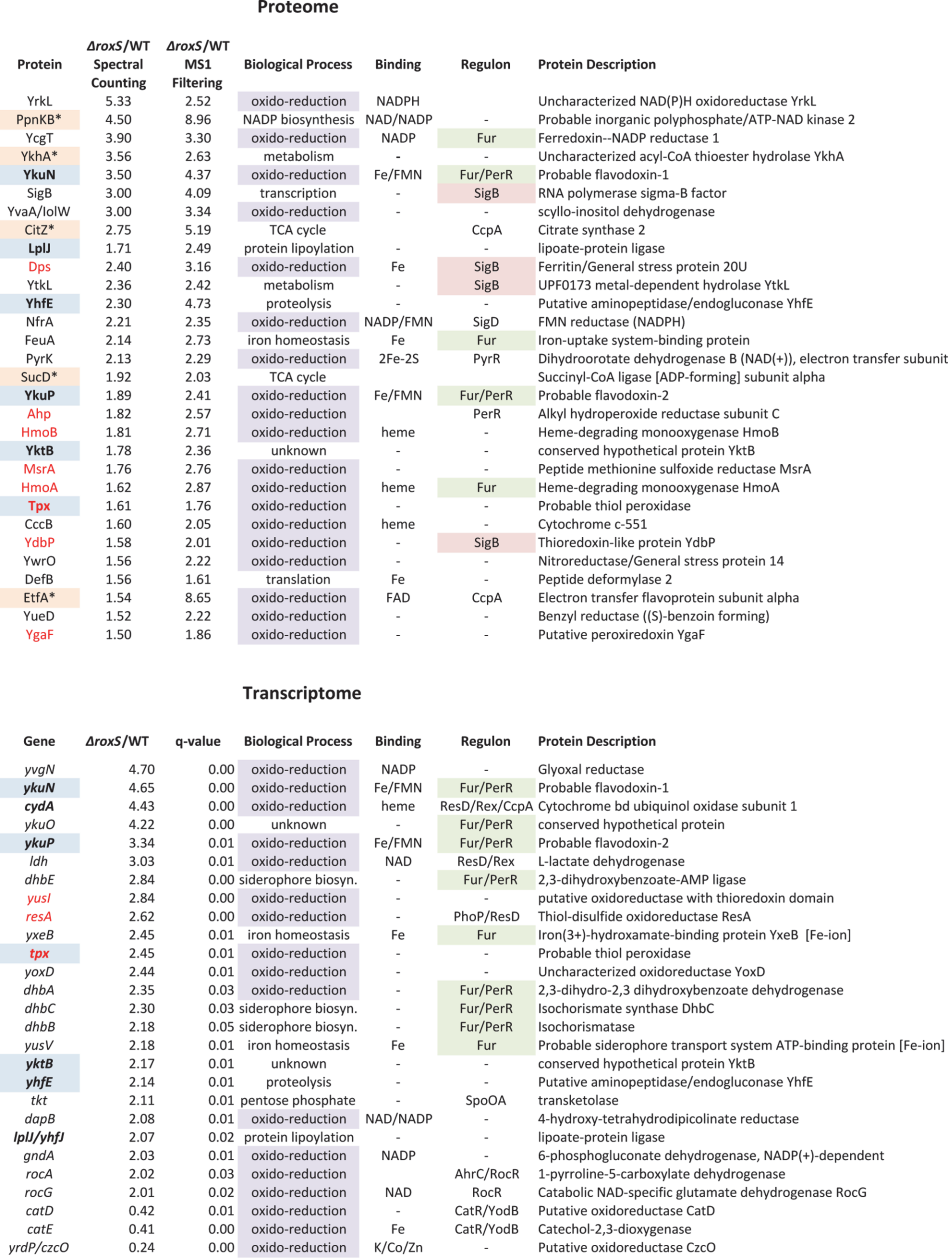
Selected candidates from proteome and transcriptome analyses of *ΔroxS strain*. Candidates found in both proteome and transcriptome are in bold type and highlighted in blue. Potential direct targets are marked with an asterisk and highlighted in beige (note for *sucD*, potential target is first gene in operon *sucC*). Candidates involved in oxido-reduction reactions are highlighted in violet. Candidates involved in oxidative stress protection are in red font. Members of the Fur and SigB regulons are highlighted in green and in pink, respectively.

To get insight into the regulatory role(s) of RoxS in *B. subtilis*, we performed both proteome and transcriptome analysis in strains lacking RoxS. We detected 1092 proteins in whole cell extracts by LC-MS/MS and identified 63 proteins with significantly increased levels in the *ΔroxS* strain compared to the WT strain (≥1.5-fold increase by two independent methods of analysis: Spectral Counting and MS1 Filtering; S1 Table). No proteins showed significantly decreased expression in the Δ*roxS* strain relative to WT. Nineteen of the up-regulated candidates (30%) had functions related to oxidation-reduction processes (Fig. 3), a significant enrichment from the 8.5% of *B. subtilis* genes associated with this Gene Ontology (GO) term (GO:0055114) genome-wide.

A number of the candidates showing increased expression in the *ΔroxS* strain (and therefore down-regulated by RoxS) are predicted to be involved in oxidative stress protection. These include the putative peroxiredoxins Tpx, AhpC and YgaF, and the thioredoxin-like protein YdbP. We also observed increased expression of the peptide methionine sulfoxide reductase MsrA, the DNA-protecting ferritin Dps and two heme-degrading mono-oxygenases HmoA and HmoB, all of which have been shown involved in resistance to oxidative stress in different *Bacilli* [35–37]. The increased expression of these proteins suggests that cells lacking RoxS are either experiencing, or behave as if they are experiencing oxidative stress, in conditions that are not normally stressful for WT cells.

Eleven of the proteins showing increased expression in the *ΔroxS* strain use prosthetic groups (NAD, FAD, FMN, heme, iron-sulfur clusters) for their oxido-reduction/electron transfer reactions (Fig. 3 and S1 Table). These include the short-chain flavodoxins YkuN and YkuP. YkuN has been shown to be capable of transporting electrons to *B. subtilis* nitric oxide synthase (bsNOS) to generate NO from arginine [38]. Interestingly, five of the proteins showing increased levels in the RoxS deletion mutant were members of the ferric uptake regulator (Fur) regulon, YkuN, YkuP, HmoA, YcgT and FeuA (iron hydroxamate binding lipoprotein), and four were members of the general stress sigma B (SigB) regulon, YdbP, Dps, YtkL (a predicted metal hydrolase) and SigB itself. One of the proteins that showed the greatest increase in expression levels was PpnKB, an inorganic polyphosphate/ATP-NAD kinase that converts NAD^+^ to NADP^+^. Although this enzyme is not directly involved in a redox reaction, it does have an influence on the cell’s levels of reducing power through the production of NADPH. The *ppnkB* mRNA is predicted to be a direct target for RoxS repression (see below).

The transcriptome analysis was performed using tiling arrays with 22 nt resolution as described previously [39]. A comparison of the RoxS deletion strain to the WT parental strain showed 46 mRNAs with increased expression levels and 48 with decreased synthesis (≥2-fold; q-value <0.05; S2 Table). Most (28/48) of the genes with decreased expression levels in the deletion strain were from the PBSX prophage, including all 12 members of the sigma factor Xpf regulon. Nine of the genes with augmented expression in the *ΔroxS* strain were members of the Fur regulon, consistent with the proteome data, although some of the genes concerned were different (Fig. 3). They include the *yxeB* and *yusV* genes, involved in the acquisition of iron, the *dhbABCE* operon involved in siderophore biosynthesis and the flavodoxin-encoding *ykuNOP* operon. YkuN and YkuP were among six candidates also identified in the proteome analysis, the others being Tpx, LplJ (lipoate protein ligase), YhfE (putative endogluconase) and YktB (unknown). We confirmed the increase in *ykuNOP* expression in the *ΔroxS* strain by Northern blot (S3A Fig.) although we suspect it may be an indirect consequence of the *roxS* deletion on Fur activity (see below). Furthermore, genes encoding a thioredoxin (*resA*) and the putative peroxiredoxin (*tpx*) also showed increased expression in the absence of RoxS. The *resA* gene encodes an extracytoplasmic thioredoxin involved in the maturation of cytochrome C, while the function of Tpx is still unknown. Two genes involved in the pentose phosphate pathway, *tkt*, encoding transketolase and *gndA*, encoding 6-phospho-gluconate dehydrogenase, also showed increased transcript levels. The pentose phosphate pathway is a major source of NADPH production in the cell for use as a reducing agent in anabolic reactions such as lipid and nucleic acid synthesis. Overall, fourteen of the up-regulated genes in the tiling array (30%) had annotated functions related to oxidation-reduction reactions (GO term 0055114), consistent with the functional enrichment seen in the proteome study (Fig. 3 and S2 Table). These data are in good agreement with a general role for RoxS in the redox state/oxidative stress response.

### RoxS inhibits initiation of *ppnKB* mRNA translation

**Figure 4:**
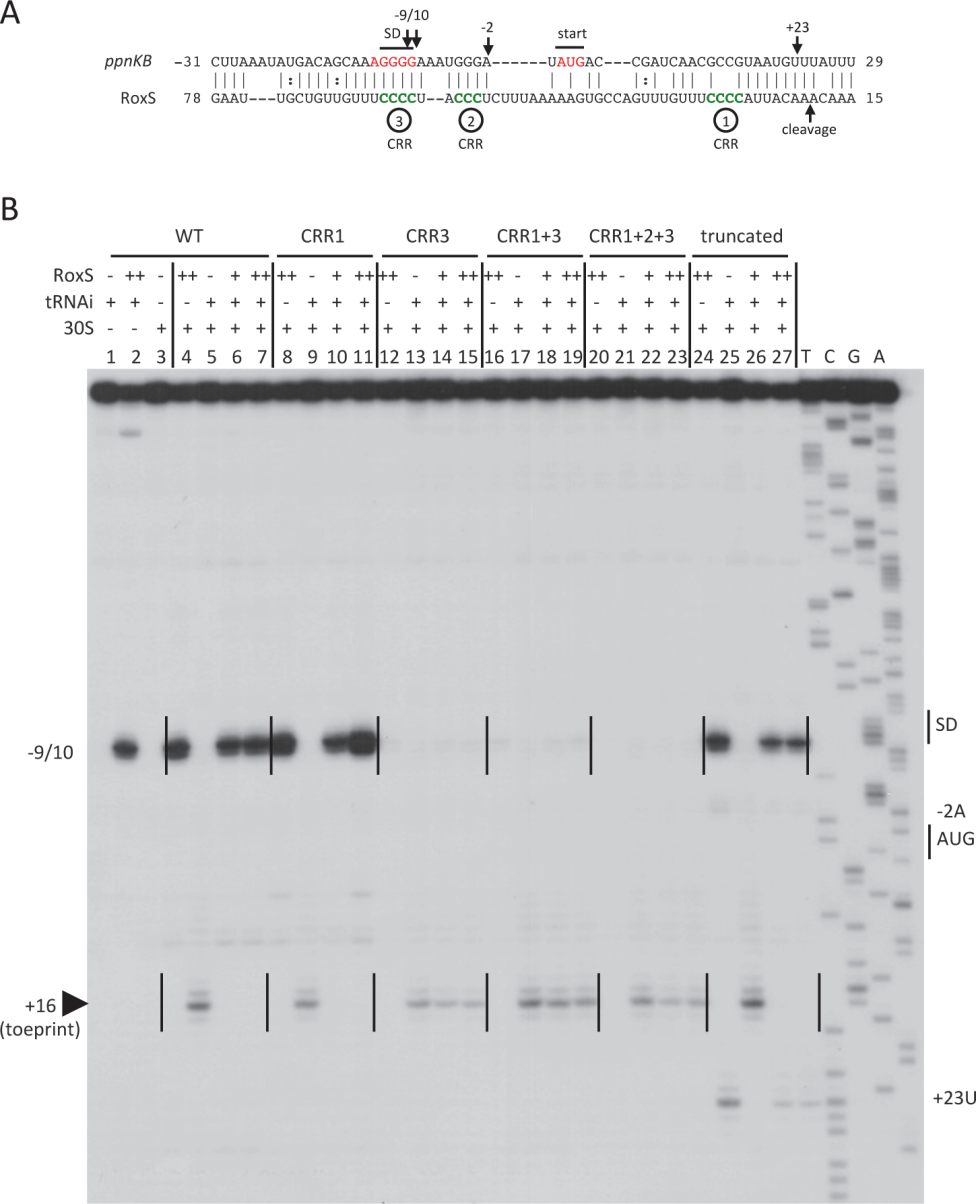
Inhibition of translation initiation complex formation on the *ppnKB* mRNA by RoxS. (A) Predicted base-pairing interactions between RoxS and the *ppnKB* mRNA by TargetRNA2 (http://cs.wellesley.edu/~btjaden/TargetRNA2/). The CRR regions 1–3 are shown in green. In the mutant forms, the four cytosine residues of CRR1 and CRR3 have been replaced by adenines and the three cytosines of CRR2 replaced by a GAA sequence. The Shine and Dalgarno (SD) sequence and AUG initiation codon (start) are indicated in red. The site of reverse transcriptase stops (−9/10, −2, +23) in the toeprint assay with full-length and truncated forms of RoxS are indicated by vertical arrows. The mapped RNase Y cleavage site in RoxS is indicated. (B) Toeprint analysis of full-length (WT) and various mutant or truncated forms of RoxS bound to the *ppnKB* mRNA. The toeprint formed by the 30S ribosomal subunit is indicated at +16 relative to the first nt of the start codon (AUG). Efficient binding of RoxS to *ppnKB* is characterized by a strong RT stop at nts −9/10. Additional RT stops observed only with the truncated form of RoxS are indicated at positions −2 and +23. The Shine and Dalgarno sequence is indicated by SD. (++) indicates addition of twice the quantity of RoxS (80 nM vs. 40 nM) as in lanes marked with (+).

Because most sRNAs base-pair to mRNA targets, we used several programs (TargetRNA2 [40], CopraRNA [41], RNApredator [42]) to predict potential direct mRNA targets of RoxS, with a particular focus on the translation initiation region. The targets suggested by CopraRNA were highly enriched for mRNAs involved in a relatively small number of cellular processes including electron transport, respiration, lipid metabolism and metal binding (S4 Fig.). The best target proposed by TargetRNA2 and RNA predator, the *ppnKB* mRNA (Fig. 4A), was consistent with this functional enrichment. Furthermore, the synthesis of the PpnKB protein was 4.5 to 9-fold increased in the *ΔroxS* strain by Spectral Counting and MS1 Filtering, respectively (Fig. 3). We therefore chose to study the RoxS-dependent regulation of *ppnkB* in more detail.

To determine whether RoxS could directly bind to the *ppnKB* mRNA to inhibit translation initiation, we tested the effect of RoxS on the formation of the ribosomal initiation complex on the *ppnKB* mRNA by toeprinting assays. Addition of 30S ribosomal subunits and initiator tRNA to the *ppnKB* transcript, showed a clear toeprint at position +16 relative to the *ppnKB* start codon (Fig. 4B; lane 5). Incubation of the *ppnKB* transcript with equimolar and higher concentrations of RoxS resulted in complete inhibition of the 30S ribosome toeprint, while a band specific to the binding of RoxS appeared at position −9/10 (Fig. 4B; lane 6 and 7). In contrast, RoxS had a much weaker effect on the formation of the initiation complex on the *ykuN* transcript (S3B Fig.) and did not show evidence for a stable interaction around the SD sequence, consistent with the fact that, despite the presence of four consecutive G-residues in the SD, it was not predicted as a target by any of the three algorithms (including the ORFs, for TargetRNA2). This experiment shows that RoxS specifically binds to the *ppnKB* mRNA and forms a stable complex that is sufficient to prevent the formation of the ternary translation initiation complex. The toeprinting assays, coupled with the fact that RoxS is not predicted to make significant interactions with any portion of the *ykuNOP* mRNA, suggest that RoxS-dependent effect on the expression this operon, observed in both the transcriptome and proteome analysis, most likely results from an indirect effect.

### C-rich region 3 is key for RoxS binding to the *ppnKB* mRNA and inhibiting translation initiation complex formation

The base-pairing interaction between RoxS and *ppnKB* predicted by TargetRNA is extensive (Fig. 4A) and includes the first three C-rich regions (CRR1-3). However, the strong reverse transcriptase (RT) stop at nt −9/10 provoked by duplex formation is close to the SD sequence, suggesting the most stable interaction is between CCR3 and the *ppnKB* ribosome binding site. However, the six nts downstream of CRR1 are identical to those downstream of CRR3, creating a 10 nt duplication (CCCCUUUGUU) in RoxS and leaving open the possibility that the two sequences were functionally redundant. We therefore performed toeprinting assays with RoxS variants where the four consecutive C-residues of CRR1 or CRR3, or both, were changed to A. These mutations are not predicted to alter the secondary structure of RoxS.

The data clearly showed that mutation of CRR3 alone abolished the ability of RoxS to bind the mRNA and to inhibit 30S ribosome binding to *ppnKB* (Fig. 4B, lanes 14–15). Conversely, mutation of CRR1 alone had no effect (Fig. 4B, lanes 10–11). RoxS mutants lacking both CRR1 and CRR3, or the three CRR’s 1, 2 and 3, behaved similarly to the CRR3 mutant in failing to interact with the *ppnKB* mRNA or to inhibit translation initiation complex formation (Fig. 4B, lanes 18–19 and 22–23). Hence, these data show that CRR3 plays the most important role in inhibition of translation initiation and that the two repeat motifs of RoxS are not functionally equivalent for the regulation of *ppnKB*.

### RoxS overexpression leads to degradation of the *ppnKB* mRNA

**Figure 5:**
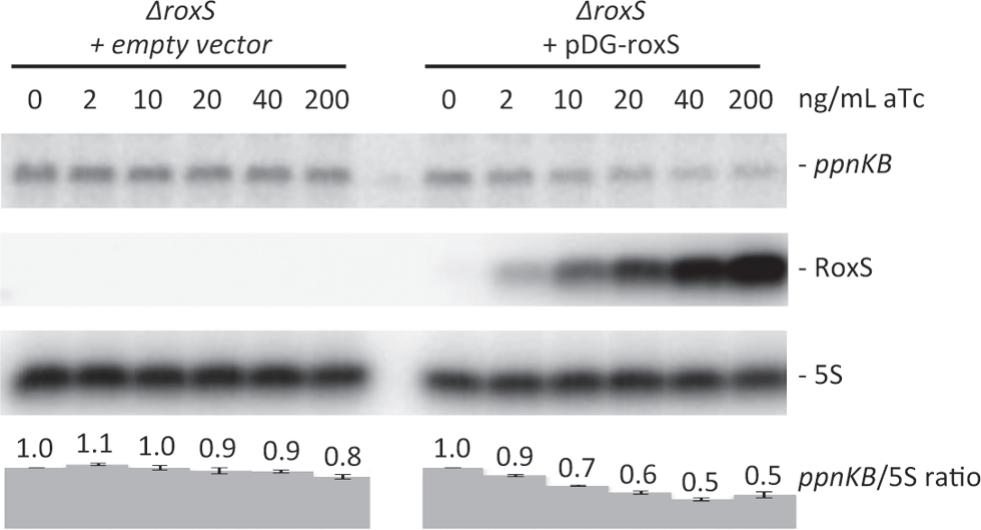
Induction of RoxS leads to decreased *ppnKB* mRNA levels. Northern blot showing decreased *ppnKB* mRNA levels upon RoxS induction. Total RNA was isolated 10 mins after addition of aTc to liquid cultures at concentrations shown and the blot was probed with oligo CC964 (S4 Table). Strains used were CCB505 (*ΔroxS* + empty vector) and CCB498 (*ΔroxS* + pDG-Ptet-roxS). 5S rRNA was probed as a loading control. *ppnKB*/5S ratios normalized to the condition without aTc are presented under the autoradiogram, with standard errors as shown.

Binding of sRNAs can have direct effects on target mRNA stability, by creating new sites for endoribonuclease cleavage, or indirect effects through the increased exposure of existing cleavage sites following translational repression. We therefore asked whether overexpression of RoxS would lead to degradation of the *ppnKB* mRNA. For these studies, we used the Δ*roxS* mutant strain transformed with a plasmid expressing RoxS from a tetracycline-dependent promoter (strain CCB498: *ΔroxS* + pDG-Ptet-roxS) or with a control plasmid (strain CCB505: *ΔroxS* + pDG-Ptet). Induction of RoxS expression with increasing concentrations of anhydrotetracylcine (aTc) in strain CCB498 caused a gradual reduction (about two-fold) in *ppnKB* mRNA levels compared to the empty vector control strain (Fig. 5), showing that RoxS affects the amount of *ppnKB* mRNA in the cell, in addition to controlling its translation.

**Figure 6:**
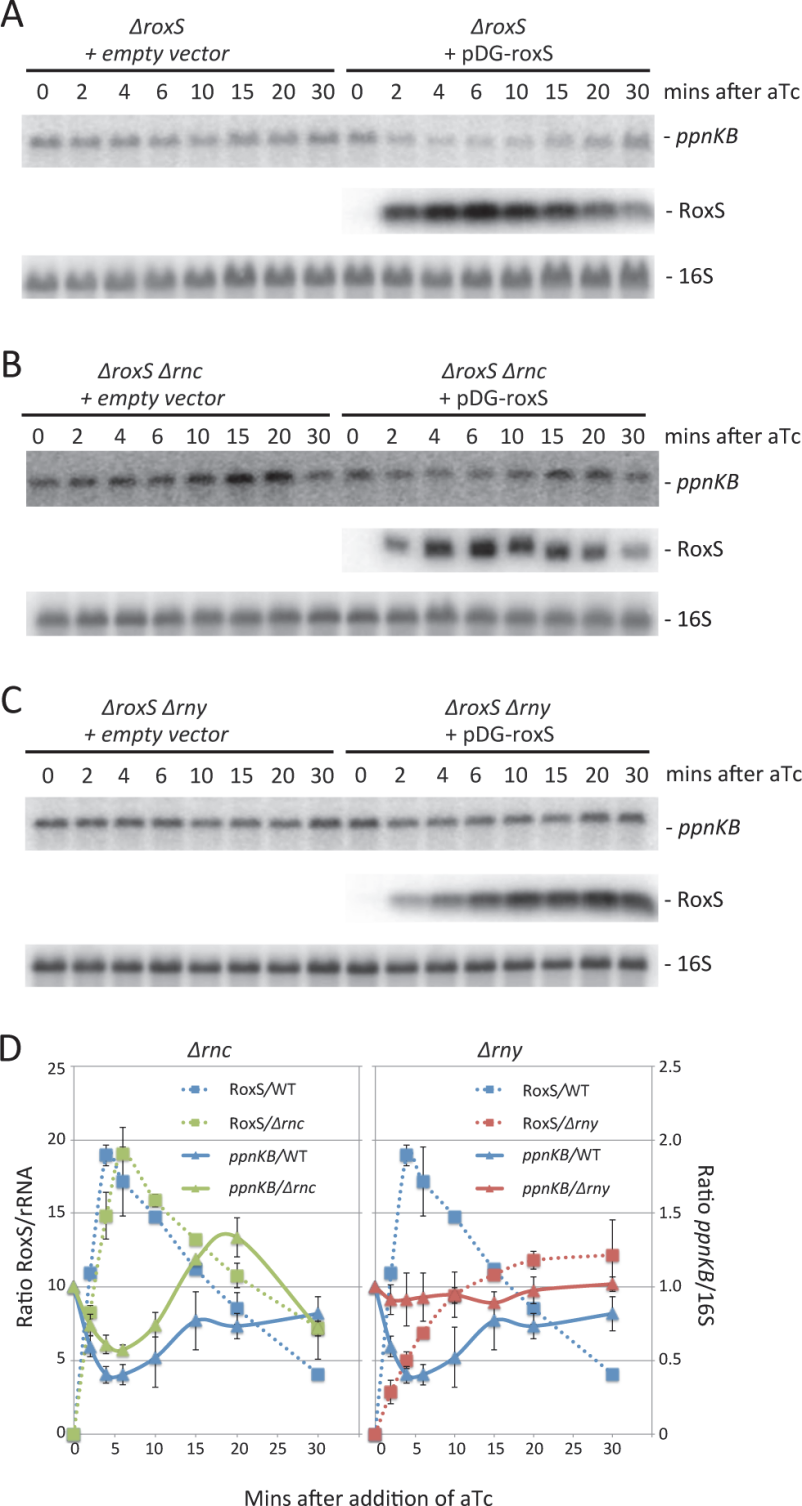
The reduction in *ppnKB* mRNA levels upon induction of RoxS expression is reversible and depends primarily on RNase Y. (A) Northern of total RNA isolated from strain CCB505 (*ΔroxS* + empty vector) and CCB498 (*ΔroxS* + pDG-Ptet-roxS) at times after the addition of 40 μg/mL aTc. The blot was re-probed for 16S rRNA (oligo CC058; S4 Table) as a loading control. (B) Same as panel A using RNase III mutant strains CCB530 (*ΔroxS Δrnc* + empty vector) and CCB531 (*ΔroxS Δrnc* + pDG-Ptet-roxS). (C) Same as panel A using RNase Y mutant strains CCB535 (*ΔroxS Δrny* + empty vector) and CCB533 (*ΔroxS Δrny* + pDG-Ptet-roxS). The RNAs isolated from strains CCB498, CCB531 and CCB533 in panels A, B and C were also run on a polyacrylamide gel and probed for RoxS. (D) Quantification of RoxS and *ppnKB* in Northern blots of strains containing pDG-Ptet-roxS. Left: WT and *Δrnc* backgrounds; Right: WT and *Δrny* backgrounds. The WT traces are the average of three experiments, and the *Δrnc* and *Δrny* traces are the average of two experiments, with standard errors as shown. *ppnKB* mRNAs were normalized to 16S rRNA and to the T0 sample (right hand Y-axis). RoxS was normalized to either 16S or 5S rRNA (left hand Y-axis).

Expression of RoxS from the pDG-Ptet vector is transient, reaching a peak about 5 mins after addition of aTc before decreasing rapidly (Fig. 6A, D), presumably due to an accumulation of the TetR repressor driven by the same promoter. We exploited this property of the plasmid to analyze whether *ppnKB* mRNA levels would recover upon shut-down of RoxS expression. Indeed, *ppnKB* mRNA levels fell to a minimum about 5 mins after induction of RoxS and were rapidly restored as RoxS levels decreased (Fig. 6A, D). The RoxS-dependent reduction in *ppnKB* levels was only slightly less efficient in a strain lacking the double-strand specific endonuclease RNase III, encoded by the *rnc* gene (Fig. 6B, D). However, it was significantly reduced in a strain lacking the single-strand specific nuclease RNase Y, encoded by *rny* (Figs. 6C, D). These results suggest that RNase Y is a key enzyme for RoxS-mediated *ppnKB* mRNA turnover, while RNase III plays a secondary role under these experimental conditions. It should be noted that RoxS is slow to shut-off in the *rny* mutant (Fig. 6C, D); we will see later that this is due to an effect of RNase Y on RoxS RNA stability.

**Figure 7:**
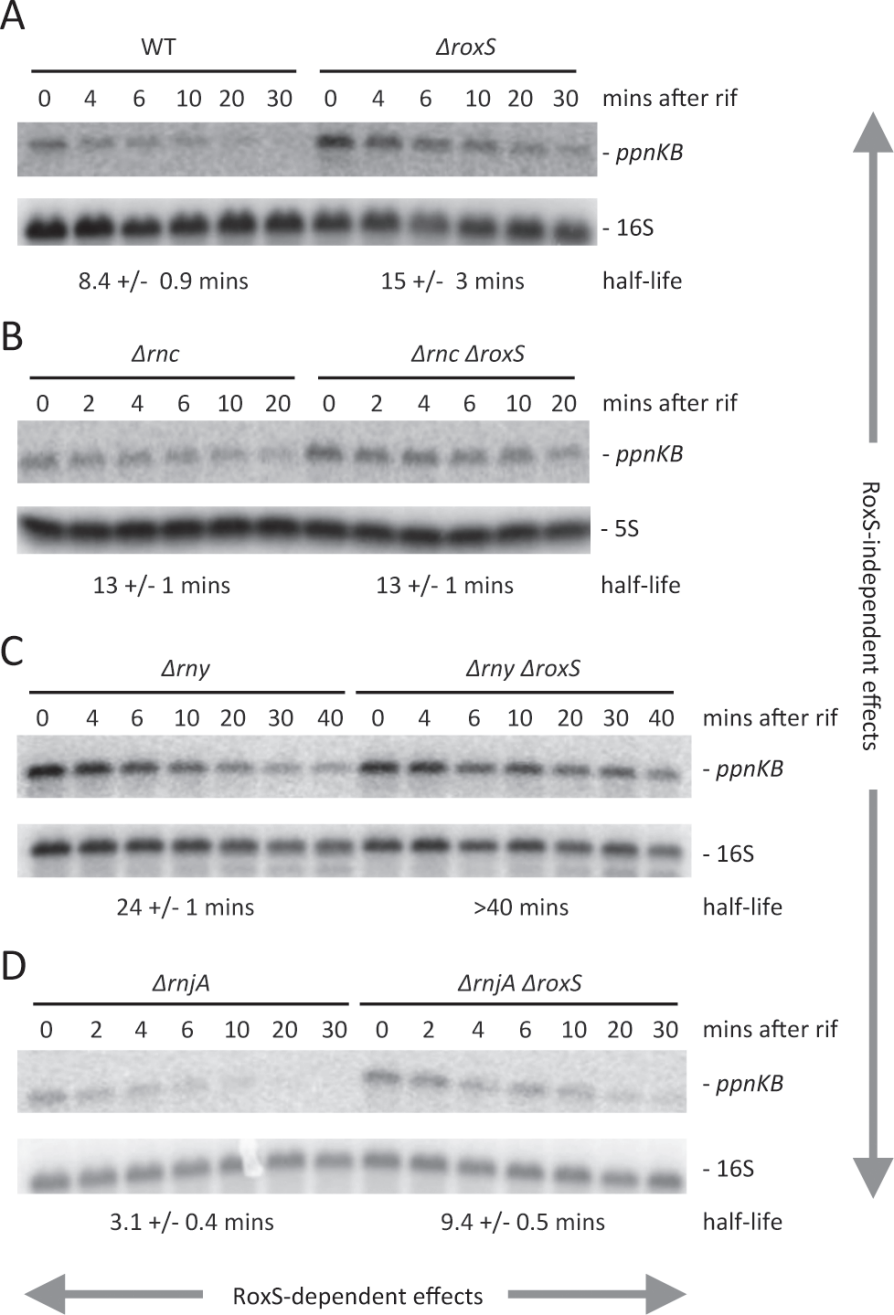
The stability of the *ppnKB* mRNA depends on RoxS, RNase III and RNase Y. (A) Northern blots of total RNA isolated from wild-type (WT) and *ΔroxS* strains (strain CCB485) probed for the *ppnKB* mRNA at times after addition of rifampicin (Rif) at 150 μg/mL. The blot was re-probed for 16S rRNA for normalization. Calculated half-lives are shown beneath the autoradiographs and are the average of 2 to 4 experiments, with standard errors as shown. (B) Same as in panel A using RNase III mutant strains CCB418 (*Δrnc*) and CCB515 (*Δrnc ΔroxS*). (C) Same as in panel A using RNase Y mutant strains CCB441 (*Δrny*) and CC558 (*Δrny ΔroxS*). (D) Same as in panel A using strains CCB434 (*ΔrnjA*) and CCB559 (*ΔrnjA ΔroxS*). RoxS-dependent effects of the different RNases are indicated by horizontal arrows and RoxS-independent effects of the different RNases by vertical arrows.

To further show that RoxS controls *ppnKB* expression at the level of mRNA stability, we measured the half-life of the *ppnKB* mRNA in WT strains and mutant strains lacking either RNase III or RNase Y under steady state conditions (Fig. 7). The *ppnKB* mRNA was stabilized about 1.8-fold in cells lacking RoxS (8.4 vs. 15 mins half life, respectively in WT and *ΔroxS* strains), consistent with a role for RoxS in controlling *ppnKB* mRNA stability (Fig. 7A). In the absence of RNase III, a similar increase in *ppnKB* stability was seen, but was not further amplified by the additional deletion of *roxS* (Fig. 7B). The simplest explanation is that RNase III and RoxS collaborate to degrade a portion of *ppnKB* transcripts; the lack of either component, or both, leading to a similar increase in *ppnKB* mRNA stability.

The *ppnKB* mRNA was also significantly stabilized (8.4 vs. 24 mins) in the *Δrny* mutant compared to the WT strain (Fig. 7C), consistent with a role for RNase Y initiating the degradation of the *ppnKB* mRNA. In this case, however, further deletion of *roxS* had an additional stabilizing effect (24 vs. >40 mins half life, respectively). This suggests the existence of a RoxS-mediated *ppnKB* turnover pathway that is independent of RNase Y and that inactivation of both pathways are required for maximal stabilization of *ppnKB*. We propose that the second pathway is the RoxS/RNase III dependent pathway described above. The data also indicate an effect of RNase Y that is independent of RoxS (15 mins vs. >40 mins half-life, respectively, in *ΔroxS* vs. *Δrny ΔroxS* strains), consistent with a role for RNase Y in the non-regulated turnover of the *ppnKB* mRNA.

Interestingly, the *ppnKB* mRNA was highly unstable in a strain lacking the 5′-3′ exoribonuclease RNase J1 (Fig. 7D) and this destabilization was attenuated upon deleting RoxS (3.1 vs. 9.4 mins half-life, respectively, in *ΔrnjA* vs. *ΔrnjA ΔroxS* strains). Data presented in the next section will shed light on this phenomenon. Globally, our data provide an illustration of the complex interplay between ribonucleases involved in the turnover of the *ppnKB* mRNA, both dependent and independent of RoxS-mediated repression.

### Evidence for two pathways of RoxS turnover

**Figure 8:**
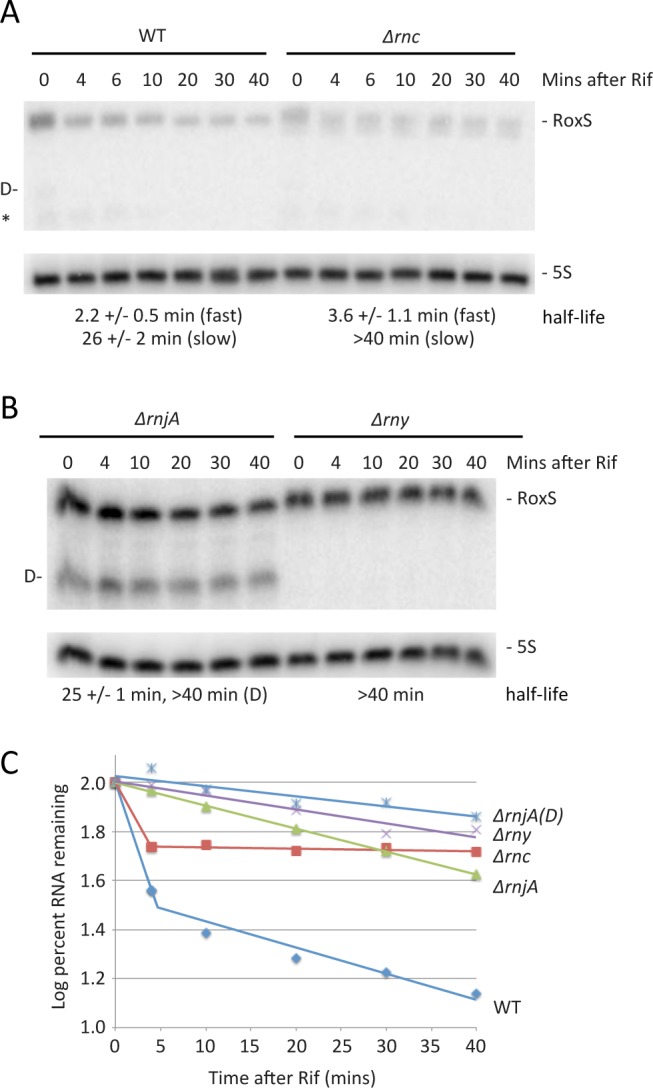
Two pathways of RoxS RNA turnover. (A) Northern blot of total RNA isolated at times after the addition of rifampicin (Rif) probed for RoxS in wild-type (WT) and *Δrnc* cells (strain CCB418). Membranes were reprobed for 5S rRNA for normalization. Half-lives are given under each blot, with standard errors as shown. In strains that have bi-phasic RNA degradation curves (see text), separate half-lives are given for the fast and slow phases of decay. The cleaved form of RoxS is labeled D on the left side of the autoradiogram. The origin of the species labeled with an asterisk is unknown. (B) Same as in panel A using strains CCB434 (*ΔrnjA)* and CCB441 (*Δrny*). (C) Graph of representative RNA decay curves showing the log percent RNA remaining versus time after rifampicin addition.

We also analyzed the importance of the three main ribonucleases in the degradation of the RoxS sRNA. The half-life of the chromosomal copy of RoxS was first measured in WT cells and in cells lacking either RNase III, RNase Y or RNase J1. In WT cells and in cells lacking RNase III, RoxS showed bi-phasic RNA degradation upon transcription arrest with rifampicin (Fig. 8A, C), suggesting that two populations of this sRNA exist *in vivo*. The simplest interpretation is that these populations represent free RoxS or RoxS bound to its targets. While the half-life of the rapidly decaying population was similar in both strains, the slowly decaying population was strongly stabilized in the *Δrnc* mutant (Fig. 8C). Because of its specificity for double-stranded RNA, it is most likely that the stabilisation of the slowly decaying phase represents stabilisation of RoxS molecules that are hybridized to its mRNA targets. In cells lacking RNase J1 (*ΔrnjA*), full length RoxS was stabilized compared to the WT strain, but in addition a very long-lived degradation/processing intermediate was detected (Fig. 8B, C). This intermediate was not detected in the absence of RNase Y and the full-length RoxS had a much longer half-life in the *Δrny* mutant strain (Fig. 8B, C). Using primer extension, we mapped the 5’ end of the short RoxS fragment to nt +20 of RoxS (S5 Fig.). Together these results suggest that RNase Y initiates RoxS turnover by cleaving around nt +20 (Fig. 1) and RNase J1 degrades the downstream cleavage product, in addition to having some activity on the full-length RNA.

Because a small amount of RNase Y-cleaved RoxS was visible in WT cells (Fig. 8A), we asked whether this truncated form was functional and might contribute to regulation. We cloned a 5′ truncated version of RoxS beginning at nt 20, called RoxS(Y), into the plasmid vector pDG-Ptet. In a manner similar to full-length RoxS, aTc induction of RoxS(Y) resulted in a rapid and efficient reduction in *ppnKB* levels, which then recovered as RoxS(Y) levels fell (S6A-S6B Fig.). Thus the truncated form of RoxS that accumulates in an RNase J1 mutant is fully functional and may explain the RoxS-dependent destabilization of *ppnKB* in the absence of RNase J1 (Fig. 7D). When tested in the toeprinting assay, the truncated RoxS species formed a more extensive hybrid than the full-length sRNA with the *ppnKB* mRNA, indicated by additional reverse transcriptase stops around nt −2 and nt +23 (Fig. 4B, lane 24). The short form was equally efficient as the full-length RoxS in inhibiting *ppnKB* translation initiation complex formation at the concentrations tested.

### RoxS forms a duplex with the ribosome binding site of *ppnKB* mRNA and creates RNase III cleavage site

**Figure 9:**
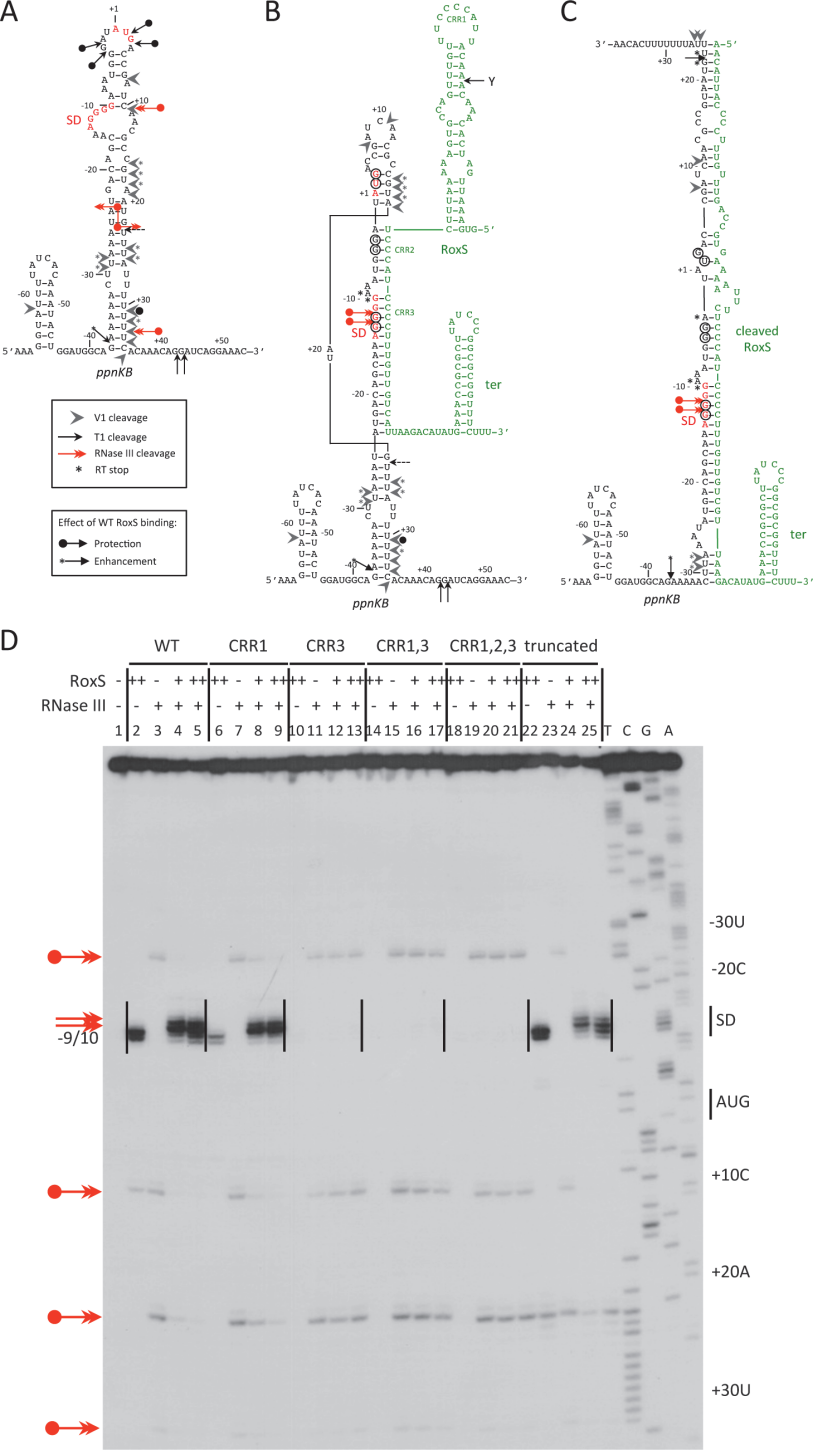
RNase III cleaves the *ppnKB*/RoxS duplex *in vitro*. (A) Summary of structure probing experiments showing proposed secondary structure around the translation initiation site of the *ppnKB* mRNA. The Shine and Dalgarno (SD) sequence is shown in red. The legend for the different cleavages is given under the schematic. (B) A proposed duplex formed between *ppnKB* (black) and full-length RoxS (green). The *ppnKB* Shine and Dalgarno sequence is shown in red. The legend for the different cleavages is the same as panel A. The CRR regions 1–3 of RoxS are indicated. The sites of protection from RNase T1 cleavages at −12/13, −3/4, and +2/3 upon duplex formation are encircled (S7 Fig.). RoxS induced RT stops are marked with an asterisk. (C) Proposed duplex formed between *ppnKB* (black) and truncated RoxS (green). Legend as in panel B. (D) Autoradiograph of *in vitro* RNase III cleavage assays showing sites of RNase III cleavage (double-headed red arrows) in *ppnKB* bound to full-length (WT) or various mutant or truncated forms of RoxS. The 5′ ends of primer extension products resulting from RNase III cleavage of *ppnKB* alone are identified to the right of the gel relative to the first nt of the AUG start codon (double-headed red arrows with circle). Note that cleavage sites are by convention identified by the nt immediately upstream of the corresponding primer extension product. The RT stops at positions −9/10 provoked by RoxS binding to *ppnKB* and the new RNase III cleavages at positions −12/13, seen upon duplex formation, are indicated to the left of the gel (double-headed red arrows). The Shine and Dalgarno sequence is indicated by SD. (++) indicates addition of twice the quantity of RoxS (80 nM vs. 40 nM) as in lanes marked with (+).

To characterize the interactions between *ppnKB* and RoxS and its various mutant forms, we performed structure probing experiments on the *ppnKB* mRNA using the double-strand-specific enzymes RNase V1 and RNase III, and RNase T1, which cleaves principally 3′ to unpaired guanines (Fig. 9 and S7 Fig.). The data suggested that in the absence of RoxS, the *ppnKB* mRNA folds into a long, but relatively unstable hairpin structure that extends from nt −37 to nt +34 relative to the translation initiation site (Fig. 9A). Indeed, two major RNase T1 cleavages occur 3′ to G-3 and G-4 and two lesser cleavages 3′ to nts +2/+3 in the apical loop containing the AUG initiation codon while a number of RNase V1 cleavages are located in the irregular helix (Fig. 9A and S7A-S7B Fig.; lane 2). Consistent with this model, RNase III cleaves the large irregular helix of *ppnKB* at four sites (nts −24, +10, +22 and +32), with the cleavages at −24 and +22 producing the two-nt 3’ overhang characteristic of RNase III processing (Fig. 9A and 9D; lane 3).

Binding of RoxS induces strong protection of the RNase T1 cleavages in the apical loop (G+3, G-3, G-4) and at G-12 and G-13 of the SD sequence while the cleavage at G-37 is slightly enhanced (Fig. 9B and S7A Fig.; lanes 3, 4). Concomitantly, RNase V1 cleavages are slightly enhanced at nts −29/30, +16 to +18, +24/25 and +31 (Fig. 9B and S7B Fig.; lanes 3, 4). Remarkably, all four RNase III cleavage sites are significantly reduced upon binding to RoxS while two strong adjacent cleavages appear in the *ppnKB* SD sequence at nts −12/13 (Fig. 9D; lanes 4, 5). These data suggest that the large hairpin loop of *ppnkB* undergoes a partial melting to promote basepairing interactions with RoxS, leading to the sequestration of the SD sequence. Identical changes in the RNase III cleavage patterns were observed if complex formation was performed with the truncated RoxS(Y) (Fig. 9C and 9D, lanes 22–25) or with the CRR1 mutant (Fig. 9D; lanes 6–9). However, RoxS derivatives with a mutation in CRR3 had no effect on the RNase III cleavages, showing that the mutated RNAs fail to interact with *ppnKB* (Fig. 9D). Identical conclusions were reached in the probing experiments with RNases T1 and V1 (S7 Fig.).

The proposed models for the interaction of *ppnkB* with full-length or truncated RoxS (Fig. 9B and C) take into account most of the data although we cannot completely distinguish between RoxS-dependent changes that are due to the formation of an extended RoxS/*ppnKB* duplex or due to a stabilization of existing *ppnKB* helices upon RoxS binding. However, the data unambiguously show that the CCR3 motif is responsible for the interaction with the SD sequence to prevent the formation of the translation initiation complex and to create a novel site for RNase III binding and cleavage.

### The *sucCD* operon mRNA is a direct target of the cleaved form of RoxS

**Figure 10:**
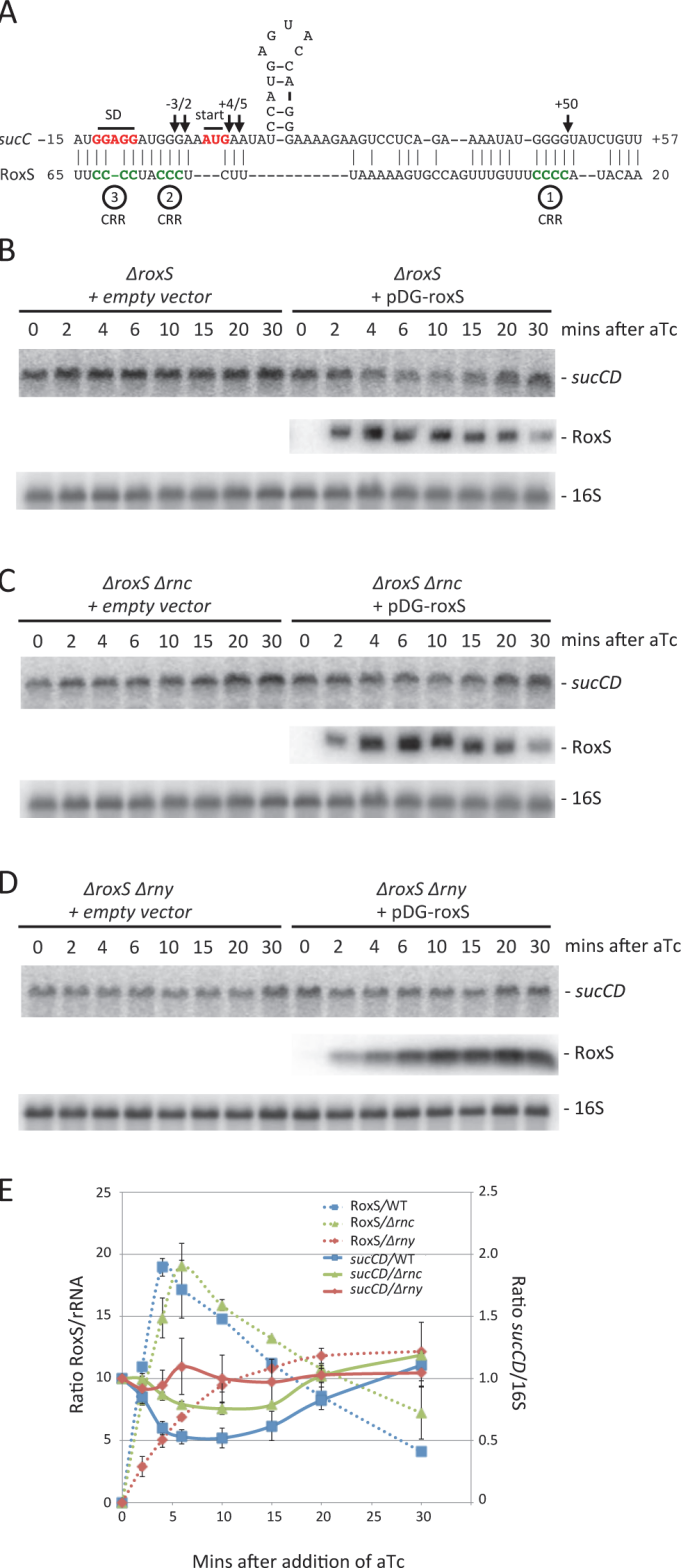
Targeted degradation of the *sucCD* mRNA upon induction of RoxS expression. (A) Predicted base pairing between RoxS and the *sucCD* mRNA by TargetRNA2 (http://cs.wellesley.edu/~btjaden/TargetRNA2/). The CRR regions 2 and 3 of RoxS are shown in green. The Shine Dalgarno (SD) sequence and AUG initiation codon (start) of *sucC* are indicated in red. (B), (C), (D) Northern blots from Fig. 5 reprobed for the *sucCD* mRNA with an oligo specific for the *sucD* portion of the bicistronic transcript (CC1408; S4 Table). (E) Quantification of RoxS and *sucCD* in Northern of strains containing pDG-Ptet-*roxS*. Legend as in Fig. 5D. The traces are the average of two experiments, with standard errors as shown.

TargetRNA2 and CopraRNA both predicted the *sucC* gene, the first cistron of the *sucCD* operon encoding the two subunits of succinyl-coA synthase, as another potential target of RoxS (Fig. 10A). Differential proteomic analysis showed a 2-fold increased expression of SucD in the mutant Δ*roxS* strain, while SucC narrowly missed the dual 1.5-fold cut-off (1.4 fold increase by spectral counting; 1.8 fold increase by MS filtering) (Fig. 3, S1 Table). Interestingly, the *sucCD* mRNA was also shown to be a target of RsaE in *S. aureus* [17,18]. We therefore probed the membranes shown in Fig. 6 for the *sucCD* mRNA to see whether its mRNA levels were affected by RoxS expression. Transient expression of RoxS by aTc addition led to a similar decrease in *sucCD* expression as was observed for *ppnKB* (Fig. 10B, E). This decrease in expression was slightly attenuated in the absence of both RNase III (Fig. 10C, E) and RNase Y (Fig. 10D, E), suggesting roles for both of these enzymes in the turnover of the *sucCD* mRNA in response to RoxS expression.

**Figure 11:**
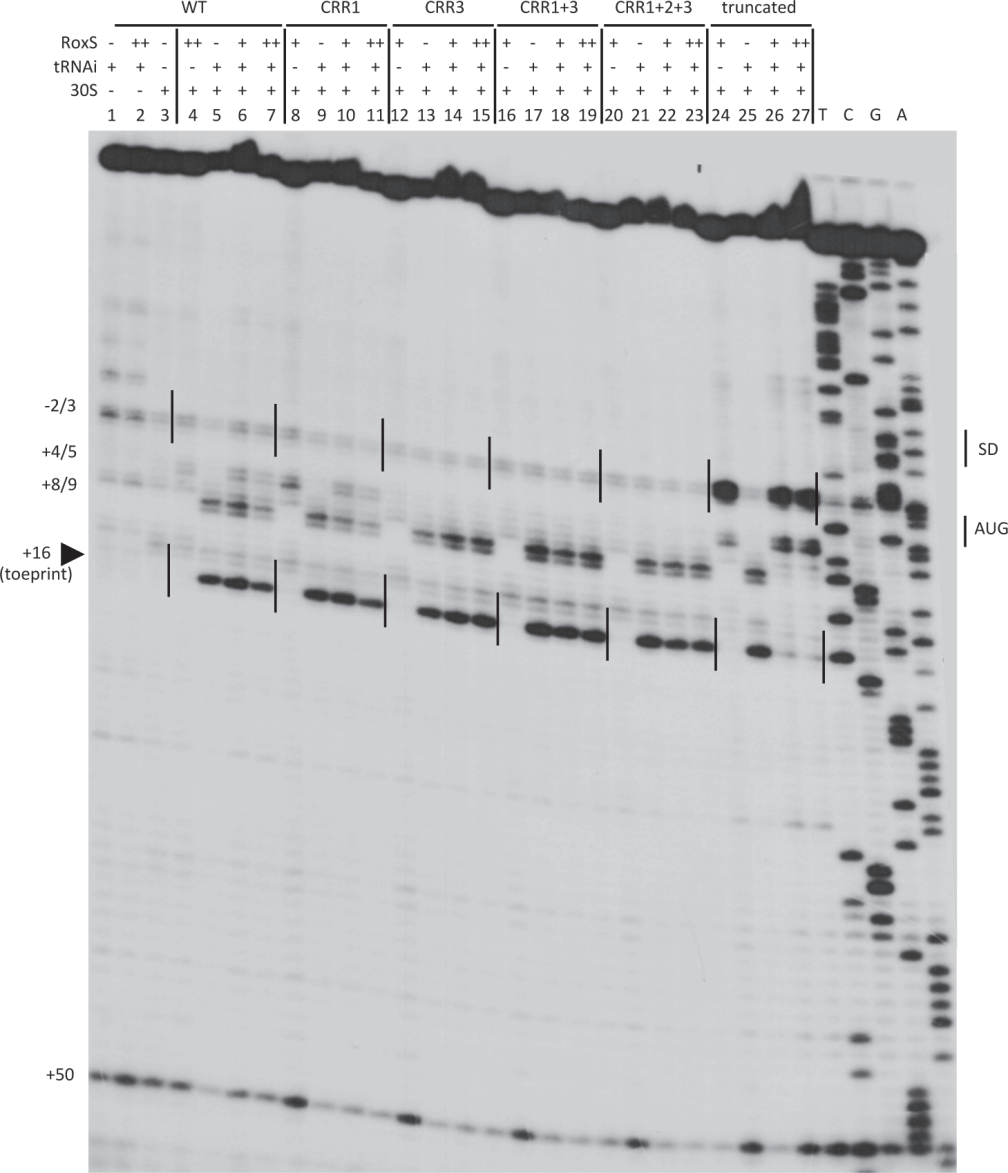
Inhibition of initiation complex formation on the *sucC* mRNA by RoxS *in vitro*. Toeprint analysis of full-length (WT) and various mutant or truncated forms of RoxS bound to the *sucC* mRNA. The major toeprint formed by the 30S ribosomal subunit is indicated at +16 relative to the first nt of the start codon (AUG). A weaker toeprint is also seen around position +8/9, which does not correspond to the functional AUG codon. Both toeprints respond similarly to RoxS binding. Weak binding of full-length RoxS to *sucC* is characterized by RT stops at nts −2/3 and +4/5. Much stronger RT stops are observed with the truncated form of RoxS. The Shine and Dalgarno sequence is indicated by SD. (++) indicates addition of twice the quantity of RoxS (80 nM vs. 40 nM) as in lanes marked with (+).

We then analyzed the effect of RoxS and its variants on the formation of the translation initiation complex formed with the *sucC* mRNA using toeprinting assays. In contrast to the *ppnKB* mRNA, RoxS binding did not prevent the formation of the initiation complex on *sucC*, even at the highest RoxS concentration (Fig. 11, lanes 6 and 7). Only a weak RT pause characteristic of RoxS binding was observed at nt +4/5 relative to the first nt of the open reading frame, indicating that RoxS did not form a stable complex with *sucC* (Fig. 11, lane 4). To our surprise, the truncated form of RoxS(Y) bound far more efficiently than RoxS to the *sucCD* mRNA, causing a very strong RT pause at position −2/−3 and an increased signal at +4/5 (Fig. 11, lane 24). As a consequence, this led to a strong and efficient inhibition of the toeprint at +16 by the truncated form of RoxS (Fig. 11, lanes 26 and 27) comparable to that seen with *ppnKB* (Fig. 4, lanes 6 and 7). This experiment suggests that processing of RoxS is necessary for regulation of *sucC* and that truncation at the 5’ end of RoxS expands the repertoire of effective targets for this sRNA.

## Discussion

### The RoxS sRNA belongs to the ResD regulon and regulates the response to NO

In this paper, we have shown that expression of the regulatory RNA RoxS/RsaE is induced by nitric oxide in both *B. subtilis* and *S. aureus*, in a mechanism that is dependent on their respective orthologous two-component systems, ResDE and SrrAB. The membrane-bound sensor protein ResE/SrrB is autophosphorylated in response to both NO and limiting O_2_ levels, and in turn phosphorylates the response regulator ResD (SrrA in *S. aureus*) [31,43]. NO and hypoxia inhibit terminal oxidases and limit the flow of electrons through the electron transport chain. It was recently suggested that the resulting accumulation of reduced menaquinones in the membrane is likely to be the trigger that activates the ResDE/SrrAB TCS [30], similar to the quinone-sensitive ArcAB TCS in *E. coli* [44]. In *S. aureus*, SrrAB is important for cell survival in the host environment and in biofilms. This system senses and responds to both NO and hypoxia, and regulates genes required for cytochrome biosynthesis and assembly, anaerobic metabolism, iron-cluster repair, and NO detoxification [43]. In *B. subtilis*, ResD is known to activate the expression of about 30 genes involved in the anaerobic respiration of nitrate, the production of cytochromes, the fermentation of pyruvate and in NO detoxification [45].

Here, we have studied in more detail the regulatory functions of *B. subtilis* RoxS, which further expands the regulatory impact of ResD. Interestingly, regulation by ResD is significantly more efficient as growth begins to slow down and RoxS expression is essentially completely ResD-dependent in early stationary phase (Fig. 1C). Despite the fact that a number of the surrounding genes show increased expression in the presence of diamide, the thiol stress regulator Spx had little effect on RoxS expression. This is consistent with a recent study by Rochat *et al.* that showed an effect of an *spx* deletion on the neighboring genes but not on *roxS* itself [33]. However, a significant number of genes with functions related to oxidation-reduction reactions or oxidative stress resistance showed increased expression in *B. subtilis* cells lacking RoxS. This surprising result suggests that *ΔroxS* cells are suffering from a deficit of reducing power. The derepression of many members of the Fur regulon, including the *ykuNOP* operon, in the *ΔroxS* deletion strain is consistent with this observation. Since Fur uses reduced iron (Fe^2+^) as a co-repressor, a deficit in reducing power would be predicted to lead to decreased Fur repressor activity and increased expression of these genes.

**Figure 12:**
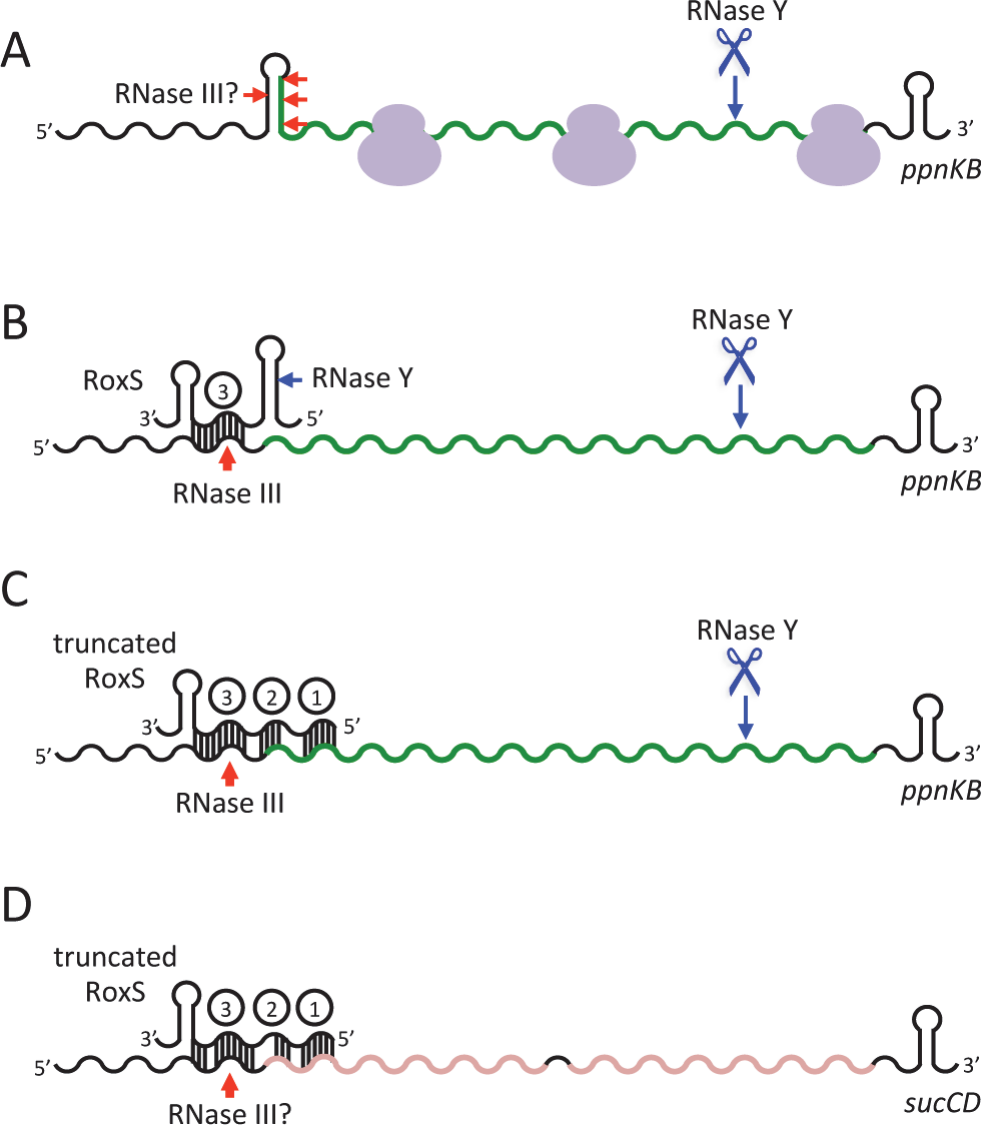
Models of RoxS regulation of the *ppnKB* and *sucCD* mRNAs. (A) RNase Y (blue scissors and arrows) can destabilize the *ppnKB* mRNA in the absence of RoxS by stochastically cleaving between passing ribosomes shown in mauve. *In vitro* data suggests there also a possibility of weak cleavage by RNase III (red arrows) in the secondary structure that can form around the translation initiation site in the absence of RoxS. (B) Binding of RoxS blocks ribosomes from initiating translation of *ppnKB*. RNase III cleaves the duplex formed between CRR3 and *ppnKB* irreversibly inactivating the mRNA for translation. The lack of ribosomes gives RNase Y free access to cleave *ppnKB*. The site of RNase Y cleavage of RoxS is shown (blue arrow). (C) The cleaved form of RoxS forms a more extended hybrid with *ppnKB* that encompasses CRR1-3 and that is as effective at regulating translation and turnover of *ppnKB* as the full-length species. *In vitro* experiments show that the hybrid between the truncated form of RoxS and *ppnKB* is also efficiently cleaved by RNase III. (D) Regulation of the *sucCD* operon requires prior cleavage of RoxS. RNase III is the principal regulator of *sucCD* turnover and we presume the cleavage site is in the RoxS/*sucCD* duplex that encompasses CRR1, 2 and 3.

We validated the direct role of RoxS in regulation of expression of *ppnKB* at both the level of translation initiation and mRNA turnover (Fig. 12). By converting NAD^+^ to NADP^+^ using ATP or inorganic polyphosphate as a phosphate donor, PpnKB shifts the metabolic balance from the production of energy from respiration, which uses primarily NADH, towards more anabolic reactions that use NADPH as a reducing agent. Expression of RoxS would therefore be expected to limit NADPH production. The increased expression of the *gndA* and *tkt* genes observed in the *ΔroxS* strain in the transcriptome experiment is consistent with this response. Tkt (transketolase) and GndA (6-phospho-gluconate dehydrogenase) are involved in the pentose phosphate pathway, a major source of NADPH synthesis in the cell. Furthermore, a recent paper has suggested that up to 40% of cellular levels NADPH come from folate metabolism in higher eukaryotes [46]. Interestingly, folate metabolism was a key target of RsaE in *S. aureus* [17,18]. We therefore suggest that one of the physiological roles of RoxS in both *B. subtilis* and *S. aureus* is to turn down expression of genes no longer required under conditions where electron transport is limited (e.g. NO stress or hypoxia), the intracellular environment is reduced and less NADPH is required. Conversely, the increased expression of *ppnKB, gndA* and *tkt* genes observed in the RoxS deletion strain is a consistent response for a cell in need of reducing power.

A direct comparison of the genes affected by the RoxS/RsaE deletion in *B. subtilis* and *S. aureus* showed only four genes in common, namely *sucD, pgk, citZ* and *yjcG/SA0873* [17,18]. Thus, although the TCA cycle is a common target for this sRNA in both species, the majority of the genes affected are different. This may reflect fundamental differences in the regulation of the expression of RoxS or its targets in relation to their respective ecological niches and circumstances in which they encounter NO. Nevertheless, it provides an interesting example of how a similarly regulated sRNA has evolved to control different targets in two distantly related bacteria.

### *B. subtilis* RoxS acts in concert with ribonucleases to regulate mRNA targets.

The toeprinting analysis showed that RoxS forms a complex with the *ppnKB* mRNA sufficiently stable to prevent ribosome initiation complex formation. Structure probing and reverse transcription stops at nts −9/10 of *ppnKB* suggested that the primary *ppnKB*/RoxS interaction occurs *via* CRR3, although we expected more extensive intermolecular pairings. However, it is likely that the 5′ stem does not easily unwind in full-length RoxS. In this regard, it is of interest that additional RT stops were seen with the truncated form of RoxS around nts −2 and +23, coherent with interactions that extend all the way to the 5′ end of the cleaved sRNA. This can be explained by the additional nucleotides in the 5′ stem that become available for base-pairing with *ppnKB* upon cleavage of the RoxS sRNA. Intriguingly, this cleavage event is also the first step in RoxS turnover. Although this processed/partially degraded form of RoxS is just as functional as the full-length species in promoting *ppnKB* mRNA turnover *in vivo*, cleavage is not necessary for inhibition of translation initiation complex formation on the *ppnKB* transcript *in vitro*. In contrast, we showed that processing of RoxS is necessary to prevent ribosome binding to the *sucCD* mRNA *in vitro*. Prediction of basepairing interactions suggest that CCR3 binds to the SD sequence in agreement with the toeprinting assays while a second stretch of nts located in the early coding region of *sucC* is complementary to CCR1 of RoxS. We propose that the key to the stabilization of the interaction between RoxS(Y) and *sucCD* is the opening of the 5’ stem of RoxS to favor basepairing with the early coding region of *sucC* (Fig. 10A). Although full-length RoxS is unable to bind efficiently to the *sucCD* mRNA *in vitro*, a specific RoxS-dependent degradation of *sucCD* mRNA was observed *in vivo* (Fig. 10) suggesting that a *trans*-acting factor, such as an RNA chaperone, might assist RoxS in the regulatory mechanism. Alternatively, sufficient quantities of the RNase Y cleaved form of RoxS may be generated *in vivo* for regulation of *sucCD*. If it were rapidly co-degraded with its target, this might explain why large quantities of the truncated form do not accumulate *in vivo*. The predicted RNase Y cleavage site in RoxS is in a region of secondary structure, unusual for this endonuclease with a preference for single-stranded RNA [47]. It is therefore possible that RNase Y benefits from its proposed association with the DEAD box helicase CshA [48] to cleave at this site. A number of other sRNAs have been shown to be processed from larger transcripts, e.g. DicF and MicL in *E. coli, ArcZ* in *E. coli* and *Salmonella enterica*, MicX in *Vibrio cholerae* [49–53]. In most of these cases, the processed form is the major form found in the cell and is considered to be the active form of the molecule. In the case of ArcZ, it was shown that while the long form was a better binder of Hfq, only the RNase E-processed species could form a Hfq-dependent complex with its target *rpoS* [54]. In the case studied here, the unprocessed form of RoxS is the major form found in cells and likely to be the principal regulatory species. Cleavage of the sRNA potentially allows it to diversify and bind to other mRNA targets, essentially creating two functional sRNAs for the price of one.

Hfq has been shown to play an important role in sRNA regulation in the Proteobacteria, but only very few sRNAs have Hfq-dependent regulation in the Firmicutes. We nonetheless addressed the question of whether deletion of the *hfq* gene had an effect on RoxS-dependent down-regulation of the *ppnKB* mRNA, but did not observe any major impact of the *hfq* mutation (S8 Fig.). This result is tempered by the observation that, in the Proteobacteria, over-expression of sRNA has sometimes been shown to by-pass the need for Hfq [54].

Because RoxS binds to the SD sequence and inhibits the initiation complex formation *in vitro*, it clearly points to a role for RoxS in inhibiting *ppnKB* translation. In addition to this regulation, *ppnKB* is controlled at the level of mRNA turnover. First, RNase III can cleave the duplex between *ppnKB* and RoxS (Fig. 12). Cleavage in the SD sequence would immediately and irreversibly render the *ppnKB* mRNA translationally non-functional. A similar mechanism has been previously proposed in the regulation of the *ompA* and *lamB* mRNAs by the MicA sRNA in *Salmonella* [55] and the *sodB* mRNA by RyhB in *E. coli* [56]. *In vitro*, RNase III cleaved the large and irregular helix in the *ppnKB* mRNA in the absence of RoxS binding, albeit weakly. We therefore cannot rule out the involvement of RNase III in the turnover of *ppnKB* in the absence of RoxS *in vivo*; however, this relatively weak structure may not be able to compete efficiently with ribosome initiation *in vivo*. Our *in vivo* data suggest that RNase Y is also an important player in turnover of the *ppnKB* mRNA in both a RoxS-dependent (Fig. 6) and RoxS-independent (Fig. 7) manner. We propose that in the absence of RoxS, RNase Y can cleave the *ppnKB* mRNA occasionally between passing ribosomes (Fig. 12A). Upon inhibition of translation by RoxS, cleavage by RNase Y would be more efficient. Although the role of RNase E in sRNA regulation is now well-established in the Enterobacteria, the equivalent enzyme in Firmicutes, which in most cases do not have RNase E, was not known. Here we show for the first time that RNase Y plays an analogous role in Gram-positive sRNA regulation.

As two endonucleases cannot generally be rate limiting for the initiation of degradation on the same RNA, the fact that the *ppnKB* mRNA half-life increases in both an *Δrnc* and *Δrny* mutant suggests that at least two different populations of the *ppnKB* mRNA exist. The simplest interpretation of the data measured under equilibrium conditions in RoxS and RNase deletion strains (Fig. 7) is that RNase III cleaves the population of *ppnKB* bound to RoxS, while RNase Y cleaves the free *ppnKB* population. However, the transient high-level RoxS induction experiment gives a different picture. Indeed, this experiment suggests that RNase Y plays the major role in RoxS-mediated *ppnKB* turnover, while the RoxS-dependent role of RNase III is relatively minor (Fig. 6). The fundamental difference between these two experiments is the amount of RoxS in the cell. We therefore hypothesize that both RNase Y and RNase III can turnover the *ppnKB* mRNA in a RoxS-dependent manner, but that the equilibrium between the two pathways depends on the amount of RoxS in the cell in ways that we do not yet fully understand.

CRR1 and CCR3 are each contained within a 10-nucleotide (nt) sequence duplication CCCCUUUGUU that can potentially base pair with the *ppnKB* SD sequence. Surprisingly, mutation of CRR1 had little effect on ribosome binding, while mutation of CRR3 essentially abolished it. Furthermore, the mutation of CRR1 and CRR3 together behaved similarly to the CRR3 mutant alone in both toeprinting assays and in the structure probing assays of the *ppnKB*/RoxS duplex, clearly showing the predominant role of CRR3 in *ppnKB* binding (Fig. 4, 9 and S7 Fig.). The structure of RoxS is most likely an important factor that explains why the two homologous sequences are not functionally redundant for *ppnKB* regulation. However, our data do not rule out a role for CRR1 in the regulation of other targets.

In addition to a whole family of sRNAs in *S. aureus* that contain CRRs [17], at least two other *B. subtilis* sRNAs, CsfG and FsrA [7,57], have CRRs in predicted single stranded regions. FsrA is a member of the Fur regulon. It contains three CRRs, two of which are separated by two nucleotides similar to the CRR2/CRR3 configuration in RoxS. CsfG also has three CRRs and, although it was characterized as an sRNA specific to sporulation in *B. subtilis*, recent evidence has suggested it may also be induced under conditions of oxidative stress [28]. These intriguing overlaps in both structure and regulation are suggestive of a functional redundancy and/or shared mechanisms of action between this class of sRNAs and their targets in Firmicutes.

## Materials and Methods

### Strains and constructs

Strains and oligonucleotides used are given in S3–S4 Tables, respectively. Strain CCB485 (*roxS::kan*) was constructed using a PCR fragment generated by re-amplifying (oligo pair CC1154/1159) three overlapping PCR fragments corresponding to upstream (oligo pair CC1154/CC1231), downstream (oligo pair CC1233/CC1159) regions of the *roxS* gene and the kanamycin resistance cassette (oligo pair CC1230/CC1232) from plasmid pDG780 [58]. The resulting PCR fragment was used to transform *B. subtilis* W168 (SSB1002)and the correct insertion verified by PCR and sequenced. Strain CCB282 (*roxS::spc*) was constructed by first constructing a plasmid where fragments corresponding to regions upstream (oligo pair RsaE-up-BamHI/RsaE-low-HindIII) and downstream (oligo pair RsaE-up-XhoI/RsaE-low-ApaI) of the *roxS* gene were amplified by PCR, cut with BamHI/HindIII and XhoI/ApaI, respectively and cloned on either side of the spectinomycin resistance cassette in plasmid pBS-Spc [59] cut with the same enzymes. The resulting plasmid was used to transform *B. subtilis* 168 (CCB281) and the correct insertion verified by PCR.

Plasmid pDG-ResDE was made by amplifying the *resDE* gene by PCR using oligo pair CC1222/1223, cutting with SphI and SalI and inserting between the same sites of pDG148 [58].

Plasmid pDG-Ptet-roxS was made from a previously made plasmid (pDG-Ptet-txpA/ratA-Plac) containing a XbaI-Pxyl/tet promoter-AatII-txpA/ratA-AatII-Plac-BstB1 construct cloned between the BstB1 and XbaI sites of pDG148 [58]. The *roxS* gene was amplified with oligos CC1216/1217 and inserted in the above plasmid cut with AatII and BstB1. The resulting plasmid pDG-Ptet-roxS permits the expression of *roxS* from its native +1 under the control of aTc. The empty vector control pDG-Ptet was made by inserting the hybridized oligos CC1328/1329 between the AatII and BstB1 sites of pDG-Ptet-txpA/ratA-Plac (above), creating a HindIII site.

Mutant derivatives of RoxS were created by PCR or overlapping PCR and cloning between the AatII and HindIII sites of pDG-Ptet. The four C’s of CRR1 were mutated to four A residues using oligo pair CC1323/1399. The four C’s of CRR3 were mutated to four A residues using overlapping oligo pairs CC1160/1327 and CC1326/1399 and reamplified with CC1160/1399. The CRR1 and CRR3 mutants were combined using oligos CC1323/1327 and CC1326/1399 and reamplified with oligos CC1323/1399. RoxS(Y) was made using oligo pair CC1398/1399. The CRR1,2,3 mutant was made by overlapping PCR using oligo pairs CC1323/1324 and CC1325/1399 and reamplified using oligos CC1323/1399. These plasmids were used for PCR amplification of templates for *in vitro* transcription of mutant RoxS RNAs.

### RNA isolation and northern blots

RNA was isolated from mid-log phase *B. subtilis* cells growing 2×YT medium either by the glass beads/phenol method described in [60] or by the RNAsnap method described in [61]. Northern blots were performed as described previously [39]. *S. aureus* strains (HG001, HG001-Δ*srrAB*) were grown to post-exponential phase by inoculating 50 ml of BHI medium with an overnight culture (1:100) at 37°C for 7 h. *S. aureus* total RNA was prepared according to the FastRNA Pro protocol (Qbiogene). After separation on agarose gels (1–2%) containing 20 mM guanidine thiocyanate, RNA was transferred onto Hybond-N+ membranes (Amersham). Detection of transcripts was done with DIG-labeled RNA according to the protocol provided by Roche.

### Tiling array and data analysis

Strains CCB281 (wild-type) and CCB282 *ΔroxS* (S3 Table) were used for transcriptome analysis. Cells were grown in 2×YT medium to OD_600_ ∼0.6. 20 mL aliquots were centrifuged and RNA was prepared by the glass beads method [60], with an additional RQ DNase (Promega) step (0.01 units/μL, 37°C for 30 mins) after the second phenol extraction. RNA concentrations were measured and duplicate samples sent to Roche/Nimblegen for labeling and analysis on second-generation (T2) tiling arrays according to the BaSysBio protocol described in [28]. The data were quantile normalized and moderated t-test p-values were computed for the difference between the Δ*roxS* and WT genetic backgrounds (R package limma [62]). The p-values were converted to q-values to account for multiple testing via control of the False Discovery Rate (FDR) (R package fdrtool [63]). The cut-off value chosen for significant effects was ≥ 2, with a Q-value of ≤ 0.05.

### Mass spectrometry based quantification: Spectral counting and MS1 label-free approaches

Strains CCB281 (WT) and CCB282 *(ΔroxS*) were used for comparative proteomic analysis. Crude extracts were obtained by trizol (Sigma) extraction, followed by acetone precipitation. Proteins (5 μg) were precipitated with 0.1 M ammonium acetate in 100% methanol, and then resuspended in 50 mM ammonium bicarbonate. After a reduction-alkylation step (5 mM DTT—10 mM iodoacetamide), proteins were digested overnight with 1/25 (W/W) of trypsin and only 3 μg of proteins was vacuum dried. The dried peptides were resuspended in 20 μL of water containing 0.1% formic acid (solvent A). The peptide mixtures were analyzed using a NanoLC-2DPlus system (with nanoFlex ChiP module; Eksigent, ABSciex, Concord, Ontario, Canada) coupled to a TripleTOF 5600 mass spectrometer (ABSciex) operating in positive mode. Each sample (5 μl; 750 ng) was loaded on a ChIP C-18 precolumn (300 μm ID × 5 mm ChromXP; Eksigent) at 2 μL/min in solvent A. After 10 min of desalting and concentration in the trap, the pre-column was switched online with the analytical ChIP C-18 analytical column (75 μm ID × 15 cm ChromXP; Eksigent) equilibrated in 95% solvent A and 5% solvent B (0.1% formic acid in acetonitrile). Peptides were eluted by using a 5%–40% gradient of solvent B for 120 min at a flow rate of 300 nL/min. The TripleTOF 5600 was operated in data-dependent acquisition mode (DDA) with Analyst software (v1.6, ABSciex). Survey MS scans were acquired during 250 ms in the 350–1250 m/z range. Up to 20 of the most intense multiply charged ions (2+ to 4+) were selected for Collision-Induced Dissociation (CID) fragmentation, if they exceeded a 150 counts per second intensity threshold. Ions were fragmented using a rolling collision energy script within a 60 ms accumulation time and an exclusion time of 15s. This so-called “Top20” method, with a constant cycle time of 1.5s, was set to high-sensitivity mode.

Data were searched against the *B. subtilis* strain 168 database from SwissProt (release 2013-11-20). The first algorithm used was Mascot (version 2.2, Matrix Science, London, UK) through the ProteinScape 3.1 package (Bruker). Variable peptide modifications allowed during the search were: N-acetyl (protein), carbamidomethylation (C) and oxidation (M). Mass tolerances in MS and MS/MS were set to 20 ppm and 0.5 Da, respectively. Two missed trypsin cleavages sites were allowed. Peptide identifications obtained from Mascot were validated with a peptide false discovery rate (FDR) of 1%. A Spectral Counting quantitative strategy was first carried on using the Mascot identification results and Proteinscape 3.1 package [64,65]. To normalize the data, a correction factor was applied for each condition according to the average total spectra number for all samples.

The Paragon algorithm (ProteinPilot package, AB Sciex) was then used to perform a second database search on the same nanoLC-MS/MS dataset and with the same decoy *B. subtilis* database. Proteins validated by Paragon at FDR 1% were submitted to a MS1 label-free quantification. For that purpose, only non-modified and unshared peptides were considered, as well as the Paragon identification confidence threshold set at 99%. Precursor ions fulfilling these criteria were transferred into PeakView package (v 2.0 with Protein Quantitation plug-in, AB Sciex) and their corresponding eXtracted Ion Chromatograms (XIC) were automatically integrated, using the following parameters: RT window ± 2 min, MS tolerance ± 0.05Da. To normalize and further process the MS1 label-free data, MarkerView software (v 1.2, ABSciex) was used: a correction factor was first applied for each condition according to the total area sum function. The statistical module from MarkerView finally allowed us to perform a Principle Component Analysis (PCA) and a Student t-test on the repeated triplicate experiments.

### Primer extension assays

Primer extension assays were performed as described previously on total RNA [59].

### Toeprinting assays

The RNA fragments were transcribed *in vitro* from PCR fragments containing the leader regions and coding sequences of *ppnkB* (oligos ppnKB T7 fw/ppnKB rev2), *ykuN* (oligos ykuN T7 fw/ykuN rev 2) and *sucC* (oligos sucC T7 fw/sucC rev2). RoxS and its mutant derivatives were made similarly using PCR fragments (oligos RsaE T7 fw/RsaE rev) amplified from their respective plasmids. The transcribed RNAs were purified by 8% polyacrylamide-8 M urea gel electrophoresis. After elution in 0.5 M ammonium acetate/1 mM EDTA buffer, the RNAs were precipitated twice with ethanol. The 5′ end-labeling of dephosphorylated DNA oligonucleotides was performed with T4 polynucleotide kinase and [γ-^32^P]ATP. Before use, RNAs were renatured by incubation at 90°C for 2 min in the absence of magnesium and salt, 1 min on ice, followed by an incubation step at 37°C for 15 mins in TMN buffer (20 mM Tris-acetate pH 7.5, 10 mM magnesium-acetate, 150 mM Na-acetate).

Toeprints were performed with *E. coli* 30S subunits. The preparation ribosomal subunits, the formation of a simplified translational initiation complex with mRNA, and the extension inhibition conditions were described according to Fechter *et al.* [66]. Standard conditions contained 15 nM RNA transcript annealed to a 5’ end-labeled oligonucleotide (ppnKB rev1, ykuN rev1: complementary to codons 16 to 22; and sucC rev1: complementary to codons 24 to 31) 250 nM *E. coli* 30S ribosomal subunits (250 nM), and 40 to 80 nM of RoxS in 10 μl of buffer containing 20 mM Tris-acetate, pH 7.5, 60 mM NH_4_Cl, 10 mM magnesium acetate, and 3 mM ß-mercaptoethanol. After 10 mins at 37°C, the initiator tRNA (1 μM) was added and the reaction was incubated for a further 5 mins at 37°C. Reverse transcription was conducted with one unit of AMV reverse transcriptase for 15 mins at 37°C.

### Structure probing experiments

RoxS-*ppnKB* mRNA formation was carried out at 37°C for 15 mins in TMN buffer containing 50 mM Hepes-NaOH pH 7.5, MgCl_2_ 5 mM, KCl 50 mM. The concentrations of RoxS or its derivatives were 40 nM and 80 nM. Enzymatic hydrolysis was performed with unlabeled *ppnKB* (0.5 pmol) in 10 μl of TMN, in the presence of 1 μg carrier tRNA at 37°C for 5 mins: RNase T1 (0.002 units, Thermo scientific), RNase V1 (1 unit, Ambion), purified *B. subtilis* RNase III (165 nM, 330 nM, 660 nM). The reactions were stopped by phenol extraction followed by RNA precipitation. The enzymatic cleavages were detected by primer extension with reverse transcriptase according to [67].

## Supporting Information

S1 Fig Chromosomal context around the *rsaE/roxS* gene in *S. aureus* and *B. subtilis*. Conserved genes are in similar colors. Promoters are indicated by black arrows, transcription terminators are shown by lollipops. Gene functions and pertinent expression patterns from Nicolas *et al.* [28] are indicated below the figure.
(TIF)

S2 Fig Alignment of RsaE/RoxS sequences from different *Staphylococci* and *Bacilli*. The RoxS sequences are boxed in blue. Likely −35 and extended −10 sequences are boxed in mauve and the putative ResD binding site is boxed in green. Positions showing 100% conservation are indicated by asterisks. Abbreviations are as follows: Sepi, *S. epidermis*; SauCol, *S. aureus* Colindale strain; Shae, *S. haemophilus*; Ban, *B. anthracis*; SauNew, *S. aureus* Newman strain; Bli, *B. lichiniformis*; Bpu, *B. pumilus*; Bsub, *B. subtilis*.
(TIF)

S3 Fig Effect of RoxS on *ykuN* expression. (A) Northern blot showing effect of RoxS deletion on *ykuN* expression. The blot was reprobed for 16S rRNA using oligo CC058. (B) Comparison of efficiency of RoxS toeprint inhibition on *ppnKB* and *ykuN* mRNAs. RoxS concentrations were 40, 80 nM for *ppnKB* and 40, 80, 150 nM for *ykuN*. The 30S toeprint +16 is labeled, as is the RT pause at −9/10 for *ppnKB*.
(TIF)

S4 Fig Functional annotation chart of potential RoxS targets produced by CopraRNA. The input RoxS sequences were from *B. subtilis, B. lichiniformis, B. cereus, B. pumilus* and *B. thuringiensis*. The enrichment score cut-off was ≥ 1.0. The X-axis represents the different functional categories, while the Y-axis shows all predicted RoxS targets with a P-value of ≤ 0.01. Functional categories belonging to the same overall group have similar colors, with darker colors representing smaller P-values.
(TIF)

S5 Fig 5’-mapping of RoxS degradation intermediate observed in strains lacking RNase J1 by primer extension. The 5’ ends corresponding to the transcriptional start site (+1) and degradation intermediate (D) are shown. Primer extension was performed with oligo CC1363. The sequence is labeled as its complement for direct read-out. Strains used were SSB1002 (WT), CCB434 *ΔrnjA* (J1) and CCB441 *Δrny* (Y).
(TIF)

S6 Fig The truncated form of RoxS is functional *in vivo*. (A) Northern blot of total RNA isolated from strain CCB498 (*ΔroxS* + pDG-Ptet-roxS) and CCB582 (*ΔroxS* + pDG-Ptet-roxS(Y)) at times after the addition of 40 μg/mL aTc. The (agarose gel) blot was probed for *ppnKB* (oligo CC964), then re-probed for *sucCD* (oligo CC1408) and 16S rRNA (oligo CC058). The RNAs were also run on a polyacrylamide gel and probed for RoxS. (B) Quantification of Northern blots shown in panel A. *ppnKB* and *sucCD* mRNAs were normalized to 16S rRNA and to the T0 sample (right hand Y-axis). RoxS was normalized to 16S rRNA only (left hand Y-axis).
(TIF)

S7 Fig Structure probing of *ppnKB* bound to wild type (WT) and various mutant/cleaved forms of RoxS. (A) Structure probing by RNase T1. The *ppnKB* mRNA was hybridized to 40 nM and 80 nM RoxS, digested by RNase T1 and assayed by primer extension using oligo ppnKB rev1. Key changes are labeled, as is the strong RT stop at −9/10 (arrow) provoked by RoxS binding to *ppnKB* mRNA (B) Structure probing by RNase V1. Legend as for panel A. Sites of RNase T1 and V1 cleavage are shown in Fig. 8A and B.
(TIF)

S8 Fig Hfq is not necessary for RoxS-dependent inhibition of *ppnKB* expression *in vivo*. (A) (A) Northern of total RNA isolated from strain CCB505 (*ΔroxS* + empty vector) and CCB498 (*ΔroxS* + pDG-Ptet-roxS) at times after the addition of 40 μg/mL aTc. The blot was re-probed for 16S rRNA (oligo CC058; S4 Table) as a loading control. (B) Same as panel A using Hfq mutant strains CCB660 (*ΔroxS Δhfq* + empty vector) and CCB661 (*ΔroxS Δhfq* + pDG-Ptet-roxS). (C) Quantification of Northern blots shown in panels A and B. *ppnKB* mRNA was normalized to 16S rRNA and to the T0 sample (right hand Y-axis). RoxS was normalized to 16S rRNA only (left hand Y-axis).
(TIF)

S1 Table Comparison of expression of *ΔroxS* and wild-type strains by proteome analysis (LC-MS/MS). The experiment was performed in triplicate. The number of replicates in which peptides were detected is given. For spectral counting only peptides with an average ≥ 5 spectra were retained. For MS1 filtering, only those with a P-value ≤ 0.05 were retained. Only proteins showing a *ΔroxS*/WT ratio ≥ 1.5 fold difference by both methods are included in the final table. The regulon to which the candidates belong is indicated. Potential direct targets are indicated with their rank and prediction program: (C) CopraRNA, (T) TargetRNA, (P) RNApredator. Bold-face type (in gene symbol lane) indicates candidates also found by transcriptome analysis.
(XLSX)

S2 Table Comparison of expression of *ΔroxS* and wild-type strains by tiling array. The experiment was performed in duplicate. Only mRNAs showing a *ΔroxS*/WT ratio ≥ 2.0 fold and a q-value ≤ 0.05 are included in the final table. The regulon to which the candidates belong is indicated. Potential direct targets are indicated with their rank and prediction program: (C) CopraRNA, (T) TargetRNA, (P) RNApredator. Bold-face type indicates candidates also found by proteome analysis. Column H: proteome data showing *ΔroxS*/WT ratio for Spectral Counting (SC)/ΔroxS/WT ratio for MS1 Filtering (MS1)/ average number of peptides in mutant/p-value for MS1 filtering. nd: not determined.
(XLSX)

S3 Table Strains used in this study. (DOC)

S4 Table Oligonucleotides used in this study. Restriction sites are in lower case letters. T7 promoter sequences are underlined.
(DOC)
