# Supplementary figures and images for: A Nitric Oxide Regulated Small RNA Controls Expression of Genes Involved in Redox Homeostasis in Bacillus subtilis
Source: PLoS Genet. 2015 Feb 2;11(2):e1004957. doi: 10.1371/journal.pgen.1004957 (PMC4409812; doi:10.1371/journal.pgen.1004957)

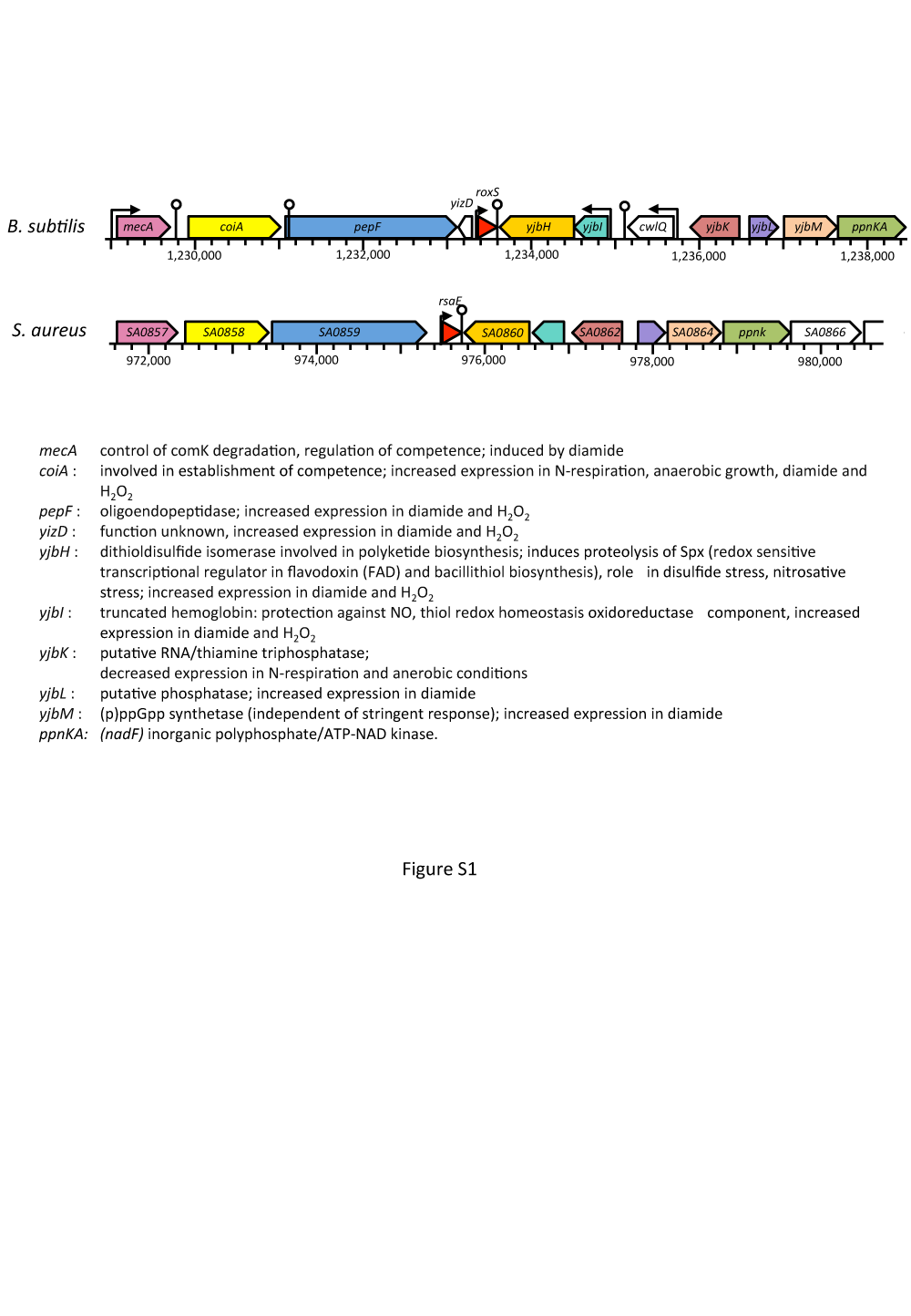

Supplement: S1 Fig — Conserved genes are in similar colors. Promoters are indicated by black arrows, transcription terminators are shown by lollipops. Gene functions and pertinent expression patterns from Nicolas et al. [28] are indicated below the figure. (TIF) [file pgen.1004957.s001.tif]

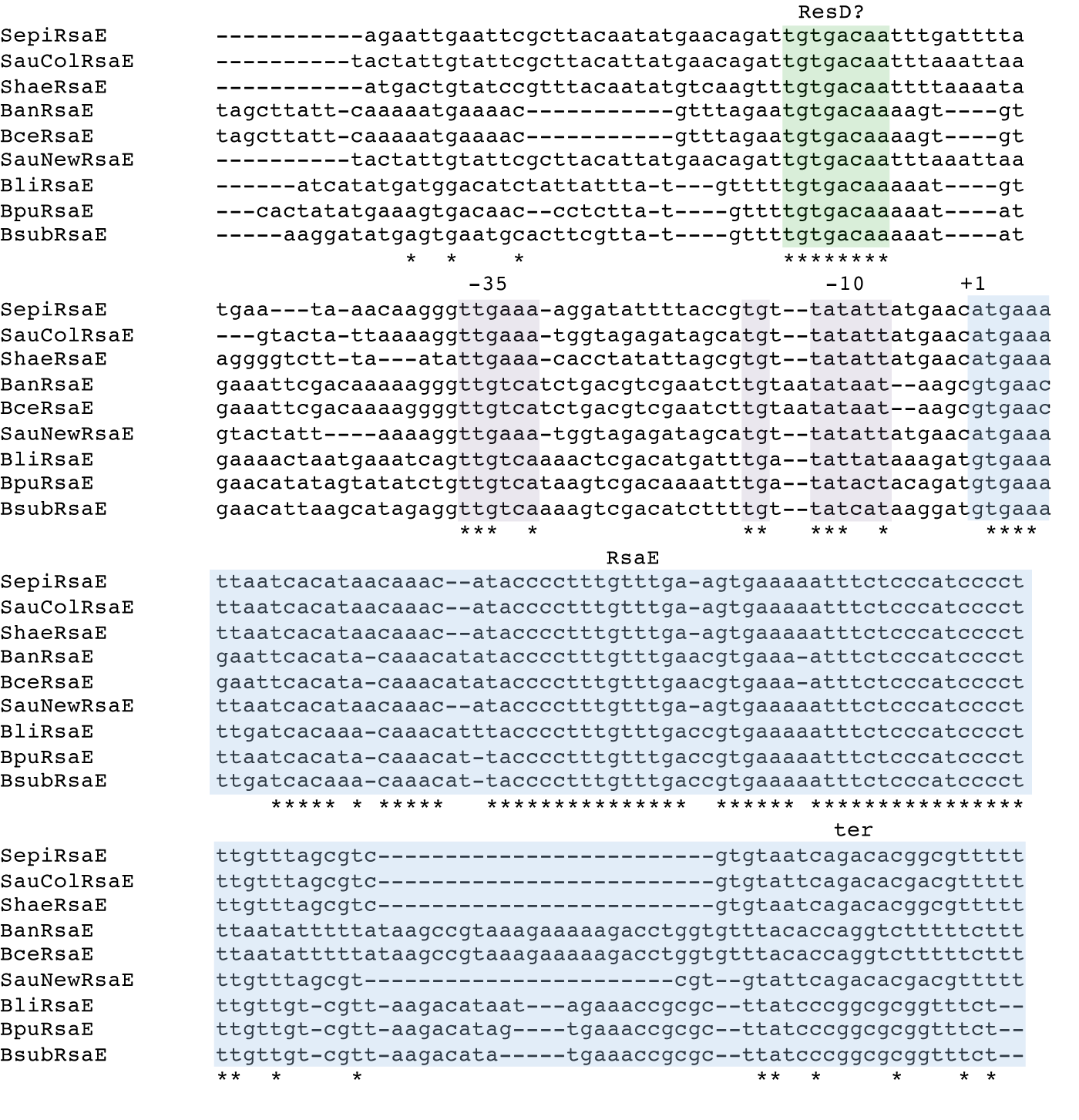

Supplement: S2 Fig — The RoxS sequences are boxed in blue. Likely −35 and extended −10 sequences are boxed in mauve and the putative ResD binding site is boxed in green. Positions showing 100% conservation are indicated by asterisks. Abbreviations are as follows: Sepi, S. epidermis; SauCol, S. aureus Colindale strain; Shae, S. haemophilus; Ban, B. anthracis; SauNew, S. aureus Newman strain; Bli, B. lichiniformis; Bpu, B. pumilus; Bsub, B. subtilis. (TIF) [file pgen.1004957.s002.tif]

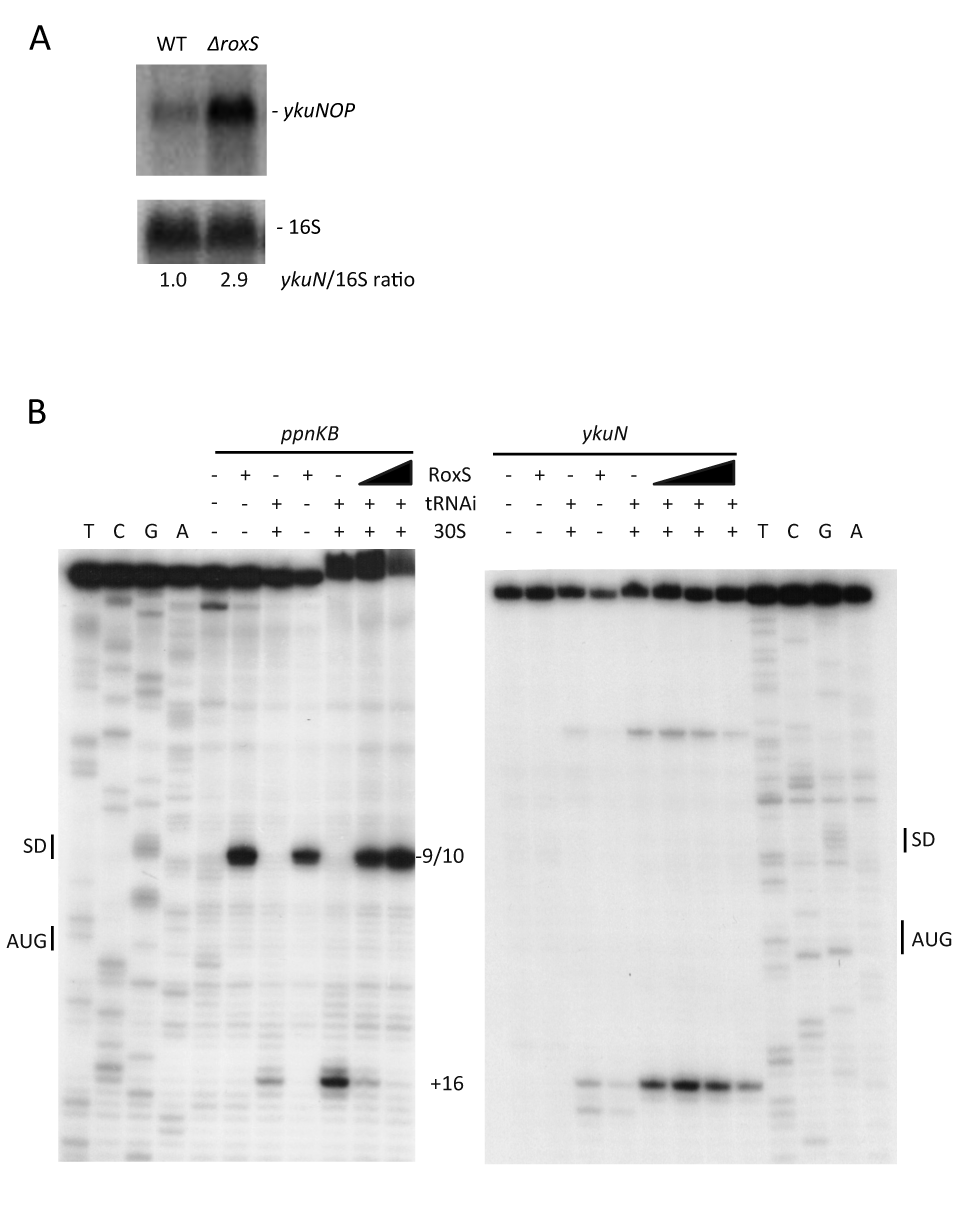

Supplement: S3 Fig — (A) Northern blot showing effect of RoxS deletion on ykuN expression. The blot was reprobed for 16S rRNA using oligo CC058. (B) Comparison of efficiency of RoxS toeprint inhibition on ppnKB and ykuN mRNAs. RoxS concentrations were 40, 80 nM for ppnKB and 40, 80, 150 nM for ykuN. The 30S toeprint +16 is labeled, as is the RT pause at −9/10 for ppnKB. (TIF) [file pgen.1004957.s003.tif]

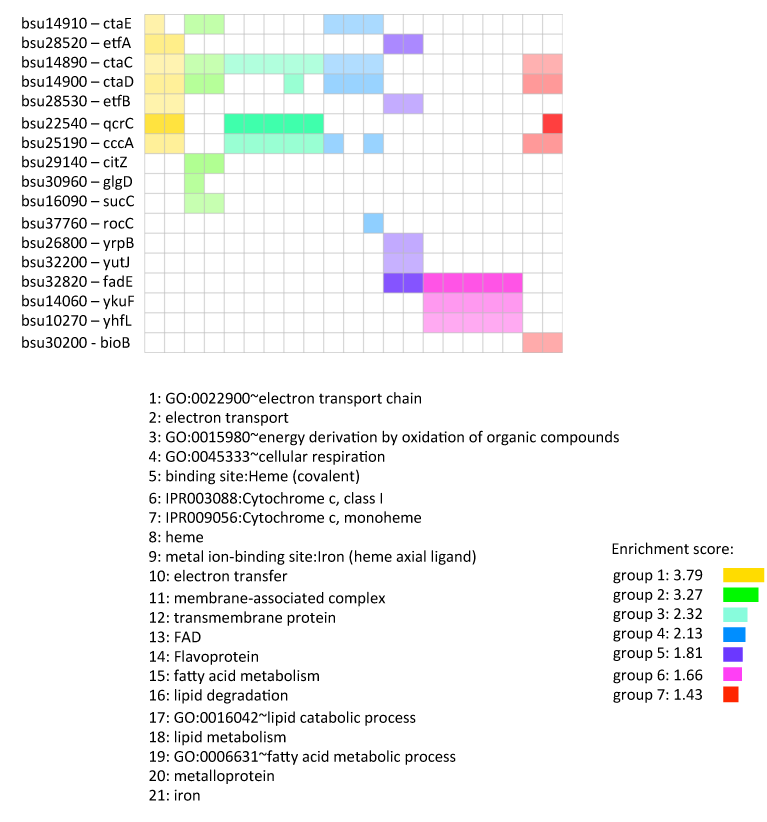

Supplement: S4 Fig — The input RoxS sequences were from B. subtilis, B. lichiniformis, B. cereus, B. pumilus and B. thuringiensis. The enrichment score cut-off was ≥ 1.0. The X-axis represents the different functional categories, while the Y-axis shows all predicted RoxS targets with a P-value of ≤ 0.01. Functional categories belonging to the same overall group have similar colors, with darker colors representing smaller P-values. (TIF) [file pgen.1004957.s004.tif]

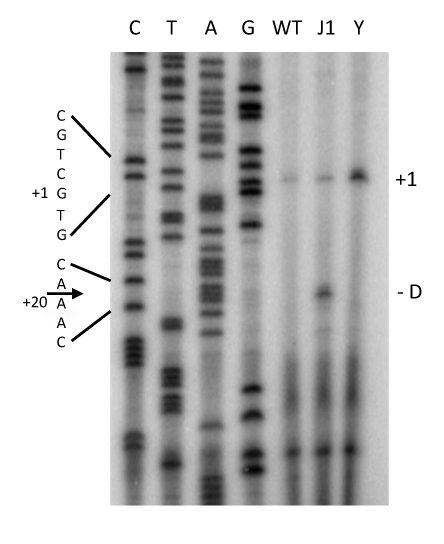

Supplement: S5 Fig — The 5’ ends corresponding to the transcriptional start site (+1) and degradation intermediate (D) are shown. Primer extension was performed with oligo CC1363. The sequence is labeled as its complement for direct read-out. Strains used were SSB1002 (WT), CCB434 ΔrnjA (J1) and CCB441 Δrny (Y). (TIF) [file pgen.1004957.s005.tif]

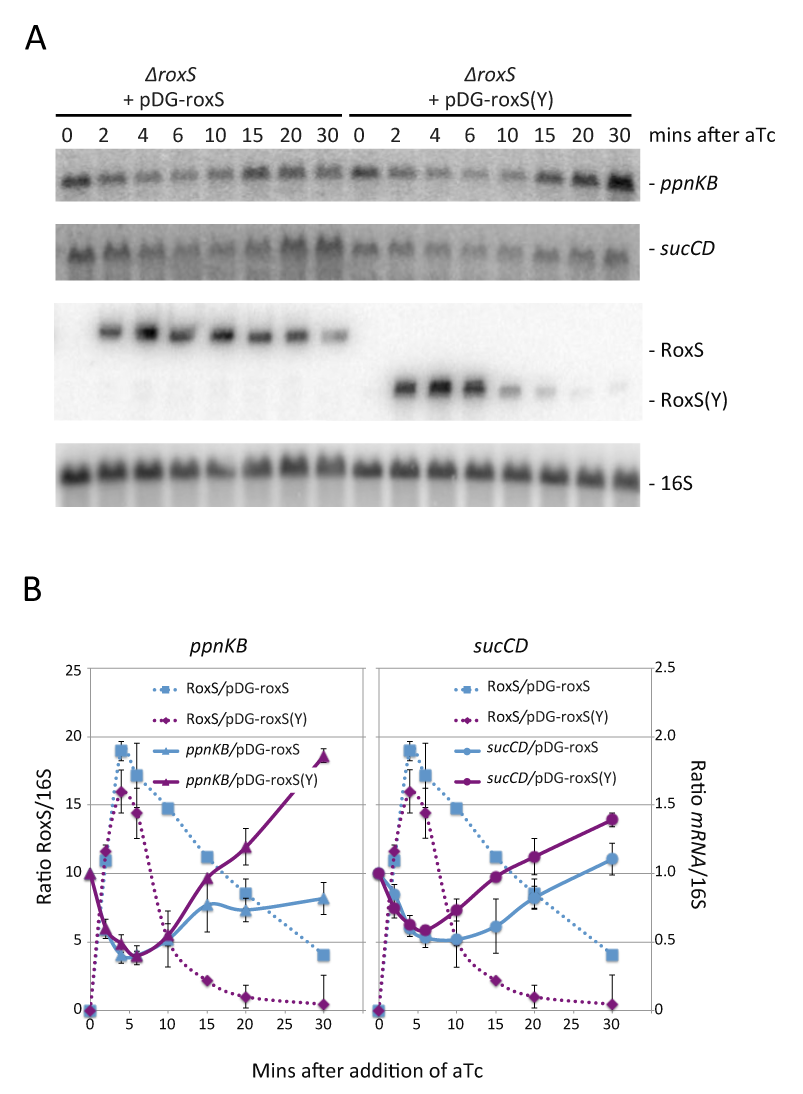

Supplement: S6 Fig — (A) Northern blot of total RNA isolated from strain CCB498 (ΔroxS + pDG-Ptet-roxS) and CCB582 (ΔroxS + pDG-Ptet-roxS(Y)) at times after the addition of 40 μg/mL aTc. The (agarose gel) blot was probed for ppnKB (oligo CC964), then re-probed for sucCD (oligo CC1408) and 16S rRNA (oligo CC058). The RNAs were also run on a polyacrylamide gel and probed for RoxS. (B) Quantification of Northern blots shown in panel A. ppnKB and sucCD mRNAs were normalized to 16S rRNA and to the T0 sample (right hand Y-axis). RoxS was normalized to 16S rRNA only (left hand Y-axis). (TIF) [file pgen.1004957.s006.tif]

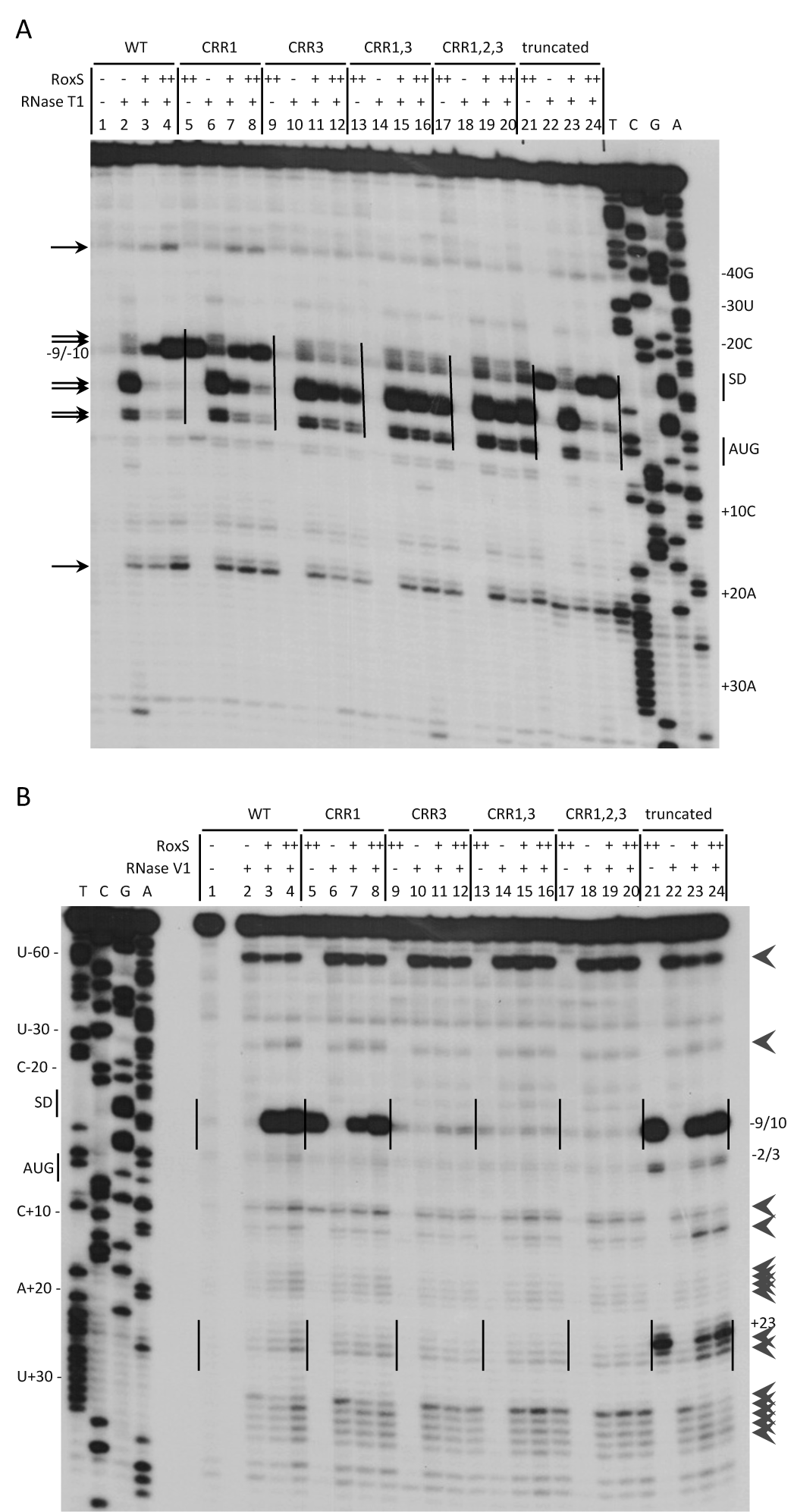

Supplement: S7 Fig — (A) Structure probing by RNase T1. The ppnKB mRNA was hybridized to 40 nM and 80 nM RoxS, digested by RNase T1 and assayed by primer extension using oligo ppnKB rev1. Key changes are labeled, as is the strong RT stop at −9/10 (arrow) provoked by RoxS binding to ppnKB mRNA (B) Structure probing by RNase V1. Legend as for panel A. Sites of RNase T1 and V1 cleavage are shown in Fig. 8A and B. (TIF) [file pgen.1004957.s007.tif]

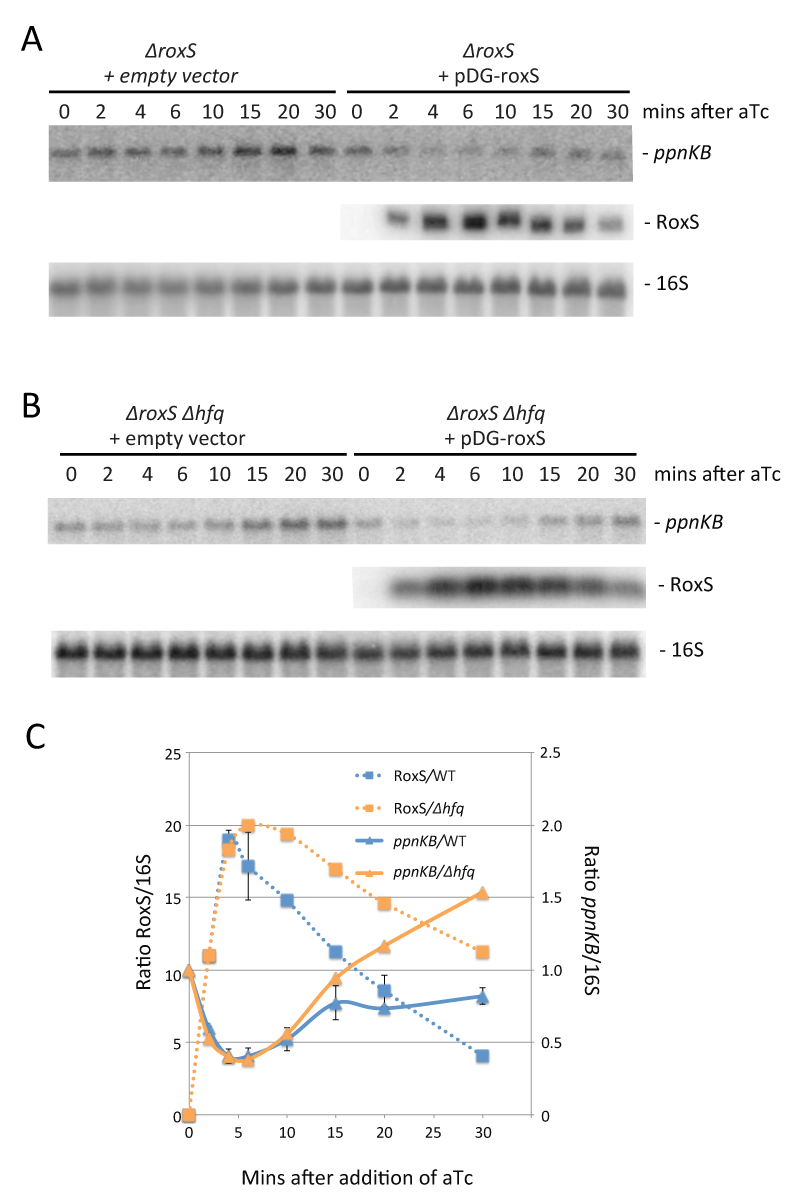

Supplement: S8 Fig — (A) (A) Northern of total RNA isolated from strain CCB505 (ΔroxS + empty vector) and CCB498 (ΔroxS + pDG-Ptet-roxS) at times after the addition of 40 μg/mL aTc. The blot was re-probed for 16S rRNA (oligo CC058; S4 Table) as a loading control. (B) Same as panel A using Hfq mutant strains CCB660 (ΔroxS Δhfq + empty vector) and CCB661 (ΔroxS Δhfq + pDG-Ptet-roxS). (C) Quantification of Northern blots shown in panels A and B. ppnKB mRNA was normalized to 16S rRNA and to the T0 sample (right hand Y-axis). RoxS was normalized to 16S rRNA only (left hand Y-axis). (TIF) [file pgen.1004957.s008.tif]
