# Supplementary material for: A Nitric Oxide Regulated Small RNA Controls Expression of Genes Involved in Redox Homeostasis in Bacillus subtilis
Source: PLoS Genet. 2015 Feb 2;11(2):e1004957. doi: 10.1371/journal.pgen.1004957 (PMC4409812; doi:10.1371/journal.pgen.1004957)
Supplement: S3 Table — (DOC) [file pgen.1004957.s011.doc]

Table S3. Bacterial strains used in this study

| Strain | Genotype | Reference |
| --- | --- | --- |
| SSB1002 | W168 *trpC*+ | Lab strain |
| CCB195 | W168 + pDG148 | This study |
| CCB281 | 168 *trpC2* | Lab strain |
| CCB282 | 168 *trpC2 roxS::spc* | This study |
| CCB310 | W168 *resDE::tet* | This study/[1] |
| CCB418 | W168 *txpA -10∆ yonT::ery rnc::spc* | [2] |
| CCB434 | W168 *rnjA::spc* | [3] |
| CCB441 | W168 *rny::spc* | [3] |
| CCB485 | W168 *roxS::kan* | This study |
| CCB498 | W168 *roxS::kan* + pDG-Ptet-roxS | This study |
| CCB503 | W168 *resDE::tet* + pDG-resDE | This study |
| CCB505 | W168 *roxS::kan* + pDG-Ptet | This study |
| CCB515 | W168 *txpA -10∆ yonT::ery rnc::spc roxS::kan* | This study |
| CCB530 | W168 *txpA -10∆ yonT::ery rnc::spc roxS::kan* + pDG-Ptet | This study |
| CCB531 | W168 *txpA -10∆ yonT::ery rnc::spc roxS::kan* + pDG-Ptet-roxS | This study |
| CCB533 | W168 *roxS::kan rny::spc* + pDG-Ptet-roxS | This study |
| CCB535 | W168 *roxS::kan rny::spc* + pDG-Ptet | This study |
| CCB558 | W168 *rny::spc roxS::kan* | This study |
| CCB559 | W168 *rnjA::spc roxS::kan* | This study |
| CCB582 | W168 *roxS::kan* + pDG-Ptet-roxS(Y) | This study |
| CCB628 | W168 *spx::cm* | This study/ [4] |
| CCB629 | W168 *resDE::tet spx::cm* | This study |
| CCB630 | W168 *roxS::kan rny::spc* + pDG-Ptet-roxS(Y) | This study |
| CCB660 | W168 *roxS::kan hfq::spc* + pDG-Ptet | This study |
| CBC661 | W168 *roxS::kan hfq::spc* + pDG-Ptet-roxS | This study |
| HG001 | *S. aureus* derivative of 8325-4, *rsb*U restored RN1, *agr* positive | [5] |
| HG001*-∆srrAB* | *S. aureus* HG001 *srrAB::kan* | Gift from Tarek Msadek |

**References for strains**

1. Nakano MM, Zuber P, Glaser P, Danchin A, Hulett FM (1996) Two-component regulatory proteins ResD-ResE are required for transcriptional activation of fnr upon oxygen limitation in Bacillus subtilis. J Bacteriol 178: 3796-3802.

2. Durand S, Gilet L, Condon C (2012) The essential function of *B. subtilis* RNase III is to silence foreign toxin genes. PLoS Genet 8: e1003181.

3. Figaro S, Durand S, Gilet L, Cayet N, Sachse M, et al. (2013) Bacillus subtilis mutants with knockouts of the genes encoding ribonucleases RNase Y and RNase J1 are viable, with major defects in cell morphology, sporulation, and competence. J Bacteriol 195: 2340-2348.

4. Rochat T, Nicolas P, Delumeau O, Rabatinova A, Korelusova J, et al. (2012) Genome-wide identification of genes directly regulated by the pleiotropic transcription factor Spx in Bacillus subtilis. Nucleic Acids Res 40: 9571-9583.

5. Herbert S, Ziebandt AK, Ohlsen K, Schafer T, Hecker M, et al. (2010) Repair of global regulators in Staphylococcus aureus 8325 and comparative analysis with other clinical isolates. Infect Immun 78: 2877-2889.
