# Supplementary material for: A Nitric Oxide Regulated Small RNA Controls Expression of Genes Involved in Redox Homeostasis in Bacillus subtilis
Source: PLoS Genet. 2015 Feb 2;11(2):e1004957. doi: 10.1371/journal.pgen.1004957 (PMC4409812; doi:10.1371/journal.pgen.1004957)
Supplement: S4 Table — Restriction sites are in lower case letters. T7 promoter sequences are underlined. (DOC) [file pgen.1004957.s012.doc]

**Table S4** Oligonucleotides used in this study. Restriction sites are in lower case lettering. The T7 promoter sequence is underlined.

| Oligo | Gene | Sequence (5'-3') |
| --- | --- | --- |
| RsaE-up-BamH1 | *roxS* | CGCggatccGACTTCCTGAAAGCGGGAAGCTCAC |
| RsaE-low-HindIII | *roxS* | CCCaagcttCACATCCTTATGATAACAAAAGATGTCGAC |
| RsaE-up-XhoI | *roxS* | CCGctcgagGTGCTAAAAATGGAACGTACGTTTGG |
| RsaE-low-ApaI | *roxS* | AAAAAAgggcccGGTCAAGGCGCTTCAATGTGAC |
| CC058 | *16S* | CAGCGTTCGTCCTGAGCCAG |
| CC875 | *roxS* | GCGGTTTCATATGTCTTAACGACAACAAAGGGG |
| CC964 | *ppnKB* | CTGCAACAGACTTGTTATAGGCGGTGCTCC |
| CC1154 | *pepF* | GACCGTTATATTGACTTCCTGAAAGCG |
| CC1159 | *yjbH* | GGTCAAGGCGCTTCAATGTGACATG |
| CC1160 | *roxS* | GTCGTGgacgtcGTGAAATTGATCACAAACAAAC |
| CC1216 | *roxS* | GTCGTGgacgtcGTGAAATTGATCACAAACAAAC |
| CC1217 | *roxS* | GTCGTGttcgaaCCAAACGTACGTTCCATTTTTAGCAC |
| CC1222 | *resDE* | GTCGTGgcatgcCACGATTTGATAAAATGAAAGTAACAG |
| CC1223 | *resDE* | TAACgtcgacCGAGTAACTGGAATTTGGAAGGG |
| CC1230 | *roxS/kan* | CAAAAATATGAACATTAAGCATAGAGGTTGCCCAGCGAACCATTTGAGGTGATAGGTAAG |
| CC1231 | *kan/roxS* | CTTACCTATCACCTCAAATGGTTCGCTGGGCAACCTCTATGCTTAATGTTCATATTTTTG |
| CC1232 | *roxS/kan* | CGTACGTTCCATTTTTAGCACAAAAAGGCATCCGTCAGGATGCCGCTTGTAGTTAAAGCTTTTTAGAC |
| CC1233 | *kan/roxS* | GTCTAAAAAGCTTTAACTACAAGCGGCATCCTGACGGATGCCTTTTTGTGCTAAAAATGGAACGTACG |
| CC1323 | *roxS* | CGTGgacgtcGTGAAATTGATCACAAACAAACATTAAAAATTTGTTTGACC |
| CC1324 | *roxS* | GTGAAAAATTTCTGAAATAAAATTTGTTGTCGTTAAG |
| CC1325 | *roxS* | CTTAACGACAACAAATTTTATTTCAGAAATTTTTCAC |
| CC1326 | *roxS* | GTGAAAAATTTCTCCCATAAAATTTGTTGTCGTTAAG |
| CC1327 | *roxS* | CTTAACGACAACAAATTTTATGGGAGAAATTTTTCAC |
| CC1328 | pDG148-Ptet | CGAAaagcttGACGT |
| CC1329 | pDG148-Ptet | CaagcttTT |
| CC1339 | *fsrA* | CGCCAAGTTTTTTCGGATCTTGATC |
| CC1363 | *roxS* | GGGATAAGCGCGGTTTCATATG |
| CC1399 | *roxS* | ATATaagcttCCAAACGTACGTTCCATTTTTAGCAC |
| CC1408 | *sucD* | TCCGGGCATAATGCCGATTTTACATTC |
| HP246 | *5S rRNA* | ATCGGCGCTGAAGAGCTTAACTTCC |
| ppnKB fw | *ppnKB* | TAATACGACTCACTATAGGG ttatggccaaaggtattttatc |
| ppnKB rev1 | *ppnKB* | cgggcttgttcgttcgtttc |
| ppnKB rev2 | *ppnKB* | taaaaaagtacgcttcac |
| ykuN fw | *ykuN* | TAATACGACTCACTATAGGG aagtgatacatatgatattg |
| ykuN rev1 | *ykuN* | ttattatgaaggcaatgtcttc |
| ykuN rev2 | *ykuN* | tgaaacatggattttttc |
| RsaE T7 fw | *roxS* | TAATACGACTCACTATAG GTGAAATTGATCACAAAC |
| RsaE rev | *roxS* | AAAGAAACCGCGCCGGG |
| SucC T7 fw | *sucC* | TAATACGACTCACTATAGGAAAGCGCAGTCTATTTTAGTTTTG |
| SucC rev1 | *sucC* | GCTTCTTCTGCTGTAAAAGCC |
| SucC rev2 | *sucC* | GCCATCACATTTCCGTCGC |
